# A Polymorphism at Position 400 in the Connection Subdomain of HIV-1 Reverse Transcriptase Affects Sensitivity to NNRTIs and RNaseH Activity

**DOI:** 10.1371/journal.pone.0074078

**Published:** 2013-10-02

**Authors:** David W. Wright, Ilona P. Deuzing, Philippe Flandre, Peter van den Eede, Micheline Govaert, Laurentia Setiawan, Peter V. Coveney, Anne-Geneviève Marcelin, Vincent Calvez, Charles A. B. Boucher, Nancy Beerens

**Affiliations:** 1 Centre for Computational Science, Department of Chemistry, University College London, United Kingdom; 2 Department of Virology, ViroscienceLab, Erasmus MC, Rotterdam, The Netherlands; 3 Institut National de la Santé et de la Recherche Médicale UMR-S 943 and Université Pierre and Marie Curie, Paris, France; 4 Janssen Diagnostics BVBA, Beerse, Belgium; Burnet Institute, Australia

## Abstract

Reverse transcriptase (RT) plays an essential role in HIV-1 replication, and inhibition of this enzyme is a key component of HIV-treatment. However, the use of RT inhibitors can lead to the emergence of drug-resistant variants. Until recently, most clinically relevant resistance mutations were found in the polymerase domain of RT. Lately, an increasing number of resistance mutations has been identified in the connection and RNaseH domain. To further explore the role of these domains we analyzed the complete RT sequence of HIV-1 subtype B patients failing therapy. Position A/T400 in the connection subdomain is polymorphic, but the proportion of T400 increases from 41% in naïve patients to 72% in patients failing therapy. Previous studies suggested a role for threonine in conferring resistance to nucleoside RT inhibitors. Here we report that T400 also mediates resistance to non-nucleoside RT inhibitors. The susceptibility to NVP and EFV was reduced 5-fold and 2-fold, respectively, in the wild-type subtype B NL4.3 background. We show that substitution A400T reduces the RNaseH activity. The changes in enzyme activity are remarkable given the distance to both the polymerase and RNaseH active sites. Molecular dynamics simulations were performed, which provide a novel atomistic mechanism for the reduction in RNaseH activity induced by T400. Substitution A400T was found to change the conformation of the RNaseH primer grip region. Formation of an additional hydrogen bond between residue T400 and E396 may play a role in this structural change. The slower degradation of the viral RNA genome may provide more time for dissociation of the bound NNRTI from the stalled RT-template/primer complex, after which reverse transcription can resume.

## Introduction

Human immunodeficiency virus type 1 (HIV-1) reverse transcriptase (RT) converts the viral RNA genome to a double-stranded DNA, which subsequently becomes integrated into the host genome. RT contains multiple enzymatic activities, including both DNA-dependent and RNA-dependent DNA polymerase activities and RNaseH activity, which are required to mediate the complex process of reverse transcription [1]. RT is a heterodimer composed of 66-kDa (p66) and 51-kDa (p51) subunits. The catalytic p66 subunit is composed of two domains: the polymerase domain, containing the fingers, palm, thumb and connection subdomains, as well as the ribonuclease H (RNaseH) domain [2]. The p51 subunit has no enzymatic function, and has been proposed to provide structural support to p66.

Because of the essential role of RT in HIV-1 replication, inhibition of this enzyme is a major component of the treatment for HIV infection. There are two classes of approved drugs that target RT: the nucleos(t)ide RT inhibitors (NRTIs) and the non-nucleoside RT inhibitors (NNRTIs). NRTIs are nucleoside analogues that lack the 3′-OH group required for formation of a phosphodiester bond with the next incoming nucleotide [3], [4]. Once incorporated into the nascent viral DNA, they act as chain terminators. On the other hand, NNRTIs bind to a pocket near the polymerase active site [5], [6]. Binding results in a conformational change in RT that inhibits polymerization. Both classes of drugs have been successfully used as key components of highly active antiretroviral therapies in combination with protease, integrase, and entry inhibitors [7]. However, as is the case with all antiviral drugs, prolonged use can lead to the emergence of drug-resistant HIV variants. The virus can become resistant to NRTIs through two mechanisms [8], [9]. Increased discrimination against the nucleoside analogue can prevent its incorporation. The polymerase domain mutations M184V, K65R and Q151M mediate resistance by the discrimination mechanism [10]–[12]. Alternatively, the ability to excise and remove chain terminators from blocked DNA chains may be enhanced, allowing DNA synthesis to resume [13]. The polymerase domain mutations M41L, D67N, K70R, L210W, T215F/Y, and K219Q/E, typically referred to as thymidine analogue resistance mutations (TAMs) are selected during treatment with zidovudine (AZT) [14], and mediate resistance by an excision mechanism. The selection of drug resistance mutations can lead to a reduction in viral replication [15], [16]. For instance, M184V is selected during treatment with lamivudine (3TC) and emtricitabine (FTC) [17], which changes the well-conserved YMDD catalytic domain, resulting in reduced polymerase activity of the RT enzyme [18]–[20]. Resistance to NNRTIs occurs as a result of mutations that decrease binding of the NNRTI, for example by mutations K103N or Y181C [21], [22].

Until recently, almost all clinically relevant NRTI and NNRTI resistance mutations were found in the polymerase domain of RT. Lately, an increasing number of mutations in the connection and RNaseH domains of RT have been shown to play a role in resistance [23]–[28]. To further explore the role of these domains, we analyzed the complete RT sequence of HIV-1 subtype B patients failing therapy. Position A/T400 is polymorphic in untreated individuals, however T400 occurs more frequently in patients failing therapy. Previous studies reported the association of T400 with NRTI resistance in HIV-1 subtype B [28], [29] and subtype AE [30], [31] and C [32]. Here we report that T400 also mediates resistance to the NNRTIs nevirapine (NVP) and efavirenz (EFV). Moreover, we explored the molecular mechanisms involved in resistance and show that it reduces RNaseH activity. Computer simulations provide a powerful tool that allows us to elucidate the atomistic origins of such changes in protein function [33]. Here we apply molecular dynamics simulations techniques to model the effects of the alanine to threonine change at position 400 on RT structure at the atomistic level. This demonstrated that an additional hydrogen bond is formed between T400 and E396 that changes the conformation of the RNaseH primer grip region. As proposed previously, the slower degradation of the viral RNA genome may provide more time for dissociation of the NNRTI from the stalled RT-template/primer complex [27], [34], [35]. Consequently, the mutant enzyme can resume reverse transcription, which results in enhanced NNRTI resistance.

## Materials and Methods

### Ethics statement

This study has been approved by the Agence Nationale de Recherches sur le SIDA AC11 resistance ethics committee. Written consent was given by the patients for their information to be stored in the hospital database and used for research.

### Analysis of HIV-1 subtype B sequences

The complete RT sequences of 135 HIV-1 subtype B patients monitored at Pitié-Salpêtrière Hospital, Paris, France, were analyzed. Of these patients, 48 never received antiretroviral treatment, whereas 87 were treated with RTI and showed virologic failure with a plasma HIV-1 RNA>1000 copies/mL. The antiretroviral treatment histories of treated patients were well-characterized by reviewing their medical records. For sequencing analysis, HIV-1 RNA was purified from 1 ml of plasma after ultracentrifugation. The entire RT coding region was amplified and sequenced from aminoacid (AA) 1 to 560 [36], [37]. Genotyping was performed by using an ABI 3100 Genetic Analyser (PE Applied Biosystems), the sequences were analysed using the Sequence Navigator software (PE Applied Biosystems) and reported as AA changes with respect to the HIV-1 subtype B consensus sequence (Stanford HIV Drug Resistance database). The association between the presence of a mutation and the treatment status was determined using the Fischer's exact test. Statistical significance of the associations was determined by applying the False Discovery Rate method of Benjamini-Hochberg [38]. This method was used to adjust for multiple testing. Considering that m tests were computed and that the raw p-values (unadjusted p-values) were ordered as p_1_<p_2_<p_3_<…<p_m_. The adjusted p-values, noted p*, were given by: 
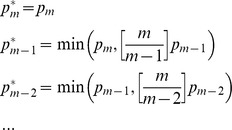



To assess significant prognostic factors of the A400T substitution a logistic regression model was used. Variables providing a p value <0.10 of the association test were potentially included in a final multivariate model.

In addition, the HIV Sequence Database in Los Alamos (http://www.hiv.lanl.gov/) and the HIV Drug Resistance Database in Stanford (http://hivdb.stanford.edu) were used to analyze the prevalence of T400 in HIV-1 subtype B sequences.

### DNA constructs

The pNL4.3-XN construct (a kind gift from Johnson Mak) was derived from the NL4.3 infectious molecular clone of HIV-1 [39] and was engineered with silent mutations that introduce XbaI and NotI restrictions sites at nucleotides 2319 and 3938, respectively [23]. This clone was shown to have wild-type levels of infectivity. The proviral clones pNL4.3-XN with mutations M184V and M41L+T215Y (TAM) were described previously [40]. For mutation of the RT gene, we used the construct pBS-RT, which contains the ApaI-SalI fragment of pNL4.3-XN (positions 2006–5785) cloned into pBluescript (Agilent Technologies). The A400T substitution was introduced using the QuickChange Site-Directed Mutagenesis Kit (Agilent Technologies), according to the manufacturer's protocol. The substitution was verified by sequencing analysis and cloned into pNL4.3-XN wild-type, M184V and TAM proviral clones using the AgeI-NotI (positions 3486–3938) restriction sites.

### Cells and viruses

293T cells were grown in Dulbecco's modified Eagle's medium containing 10% fetal calf serum (FCS) at 37°C and 5% CO_2_. For generation of the HIV-1 virus stocks, 293T cells were transiently transfected using the calcium phosphate method (CalPhos kit, Clontech), according to the manufacturers' protocol. Three days after transfection, the culture medium was centrifuged at 1,600 rpm for 10 min to remove cells, and subsequently filtered through a 0.45 µm pore-size filter. The virus stocks were stored at −80°C. Virus production was determined by measuring the capsid (CA)-p24 levels by enzyme-linked immunosorbent assay (ELISA) (Aalto).

SupT1 T cells were grown in RPMI 1640 medium supplemented with 10% FCS at 37°C and 5% CO_2_. The replication capacity of the viruses was studied in SupT1 cells. Therefore, 0.5*10^6^ cells were seeded in a 25-cm^2^ culture flask containing 5 ml of culture medium. The cells were infected with equal amounts of virus, 50 ng based on CA-p24. Virus production was monitored by measuring the amount of CA-p24 in the culture medium at several days after infection.

The MT4-LTR-EGFP cell line [41] was maintained in RPMI 1640 supplemented with 10% FCS, 0.02% gentamicin and 0.8% G418 at 37°C and 5% CO_2_.

### Drug susceptibility assays

The assay was performed as described previously [41]. Briefly, virus stocks were titrated and equal amounts of infectious virus were used to infect MT4-LTR-EGFP cells in the presence or absence of RTI. Four-fold dilutions were used for all RTIs, the range of dilutions used for each drug was a function of the IC_50_ value of the respective compound. After 3 days, EGFP expression was determined by fluorescent microscopy. The results were expressed as IC_50_ values, defined as the concentration of compound achieving 50% inhibition of the virus-induced EGFP signals as compared to the untreated virus-infected control cells. The ratio between the A400T and wild-type virus IC_50_ gives the fold-change value, as an indicator of the level of resistance mediated by the substitution. The RT inhibitors zidovudine (AZT), didanosine (ddI), stavudine (d4T) lamivudine (3TC), emtricitabine (FTC), abacavir (ABC), tenofovir (TDF), nevirapine (NVP) and efavirenz (EFV) were purchased from Toronto Research Chemicals Inc. Etravirine (ETR) is a GMP manufactured Johnson & Johnson compound.

### Virion-derived RT

293T cells were transiently transfected as described above. Three days after transfection, the culture medium was centrifuged at 1,600 rpm for 10 min to remove cells, and filtered through a 0.45 µm pore-size filter. The virions were pelleted by centrifugation at 25,000 rpm for 60 min in a Beckman SW28 rotor. Virions were resuspended in 500 µl of NP buffer (50 mM TRIS-HCl, pH 7.5, 50 mM NaCl, 0.5% NP40), and aliquots were stored at −20°C.

### Polymerase assays

Minus-strand DNA synthesis was initiated on a 51-nt HIV-1 RNA template, representing the HIV-1 primer binding site region (5′-uggaaaaucucuagcaguggcgcccgaacagggacuugaaagcga-aaguaa-3′) using a 21-nt DNA primer, complementary to the 18-nt HIV-1 PBS sequence with 3 additional nts at its 5′-end (5′-CAAGTCCCTGTTCGGGCGCCA-3′). The DNA primer was 5′-end-labeled with γ-^32^P-ATP and T4 polynucleotide kinase (NEB). For annealing, 50 ng of RNA template was incubated with 65 ng of labeled DNA primer in 15 µl annealing buffer (83 mM TRIS-HCl, pH 7.5, 125 mM KCl) for 10 min at 65°C, followed by snap-cooling on ice. Polymerization was initiated by the addition of 100 ng (based on CA-p24) of virus lysate in 40 ul of RT buffer (50 mM TRIS-HCl, pH 7.5, 50 mM KCl, 10 mM DTT, 10 µM dNTPs, 3 mM MgCl_2_). The reactions were incubated for 3, 7 and 15 min at 37°C, then stopped by the addition of formamide loading buffer, and analyzed on 12% denaturing sequencing gels. Products were quantified using the Typhoon imaging system.

### RNaseH assays

The 51-nt HIV-1 RNA template was 5′-end-labeled with γ-^32^P-ATP and T4 polynucleotide kinase (NEB). For annealing, 10 ng of labelled RNA template was incubated with 10 ng of the DNA primer in 10 µl annealing buffer (83 mM TRIS-HCl, pH 7.5, 125 mM KCl) for 10 min at 65°C, followed by snap-cooling on ice. The primer/template complex was incubated with 50 ng (based on CA-p24) of virus lysate in 40 ul of RH buffer (50 mM TRIS-HCl, pH 7.5, 50 mM KCl, 10 mM DTT, 1 mM EDTA). The reaction was started by the addition of 6 mM MgCl_2_ and incubated for 3, 7, 20 and 40 min at 37°C. The reactions were stopped by the addition of 5 mM EDTA and formamide loading buffer, and analyzed on 12% denaturing sequencing gels. Products were quantified using the Typhoon imaging system.

### Molecular dynamics computer simulations

Four HIV-1 RT structures were selected to provide the starting structures for our molecular dynamics simulations. The structures chosen contain HIV-1 RT bound to NVP (PDB: 1VRT), double stranded DNA (PDB: 2HMI), a hybrid RNA/DNA duplex based on the polypurine tract (PDB: 1HYS) and the apo enzyme (PDB: 1DLO). The sequences of the template strands of the double stranded DNA and RNA/DNA hybrid complexes are 5′-GTCCCTGTTCGGGCGCCAC-3′ and 5′-UCAGCCACUUUUUAAAAGAAAAG-3′ respectively. The later sequence represents a sequence similar to that of the HIV PPT.

Models of the NL4.3 wildtype and incorporating T400 sequences were generated via homology modelling using a combination of the VMD [42] and Deepview (formerly known as Swiss-PDBViewer) packages [43]. In each case the final model contains 556 residues in the first (p66) chain and 427 in the second (p51) chain for a total of 983 residues. Each system was solvated using a cubic box of TIP3P water molecules [44] providing a buffer of at least 14 Å distance around the protein. The systems were neutralized by the addition of Cl^−^ ions for the apo enzyme and NVP bound complexes and Na^+^ ions for those containing nucleic acids. No Mg^2+^ ions were present in any of the crystal structures and none were added. The final models (including solvent) contained approximately 180,000 atoms. The force field parameters for the inhibitor NVP were described using the General AMBER Force Field (GAFF) [45]. The protein and nucleic acid elements of all systems were described by the standard AMBER force field (ff03) [46]. Simulations for each system produced 20 ns of production simulation (following a 6 ns equilibration run) using the NAMD2 software [47] in isothermal-isobaric (NPT) conditions, with a temperature of 300K and a pressure of 1 bar. Full details of the simulation protocol and equilibration analysis are provided in the supporting information (Fig S1–S3 and Table S1 in File S1). Protein structural analysis was performed using VMD [42] and the AmberTools [48] software and nucleic acid conformations via 3DNA [49], [50].

## Results

### Increased frequency of T400 in HIV-1 subtype B individuals failing therapy

The complete RT sequence of 135 HIV-1 subtype B patients in France was sequenced to identify novel mutations in the connection and RNaseH domain of RT involved in drug resistance. The RT sequences derived from the 48 drug-naïve and the 87 RTI-treated patients were analyzed. From the treated patients, 28% received a therapy containing NRTIs (median of 5 NRTIs), whereas 72% received a therapy containing both NRTIs and NNRTIs. We identified 17 positions significantly associated with RTI exposure (Table 1). Among them, 13 were located in the polymerase domain and are known to be associated with RTI resistance (M41, K65, D67, T69, K70, L74, K103, Y181, M184, G190, L210, T215 and K219) [51]. Three other polymerase domain mutations (K43, V118, and L228) were previously reported to be associated with NRTI therapy [52]–[55]. In addition, position 400 in the connection subdomain of RT was identified (Table 1). Position 400 is polymorphic in naïve patients, alanine is present in 51%, threonine in 41%, and other residues are found in 8% of the sequences. However, in patients failing therapy, A400 is present in 22%, T400 in 72% and other residues are found in 6% of the sequences. We thus observed an increase in the presence of T400 in patients failing therapy. The relationship between position 400 and mutations in the polymerase domain was investigated. Eleven out of 16 mutations were found significantly associated with position T400 (p<0.05 in Table 2). In particular, T400 was found in 65% of the viruses harbouring mutation M184V/I, and in 57% of the viruses containing mutation T215Y/F. An association with several other TAMs, and three positions involved in NNRTI resistance (103, 181 and 190) was found, as is summarized in Table 2 (p<0.05). Further analysis was performed to determine the association of T400 with specific virologic and therapeutic parameters. We identified treatment experience (p = 0.0009) and zidovudine (AZT) (p = 0.05), abacavir (ABC) (p = 0.006) or nevirapine (NVP) (p = 0.02) exposure, presence of at least one TAM (p = 0.0006), at least 4 NRTIs received in the past (p<0.0001) and NNRTI exposure (p = 0.001) to be associated with the presence of T400. Furthermore, prior exposure to ABC (OR = 4.34, p = 0.014) and to NVP (OR = 3.66, p = 0.046) were independently associated with T400. Treatment with either ABC or NVP thus resulted in an approximate four-fold increase in the presence of a threonine at position 400.

**Table 1:** Changes in the RT sequence of HIV-1 subtype B viruses that are found with increased frequency in patients failing therapy.

			HIV-1 patients (%)	
Rank	Mutated position	Corrected p-valuea,b	Naive	Treated
1	M184	<0.0001	0	84
2	T215	<0.0001	4	70
3	M41	<0.0001	0	60
4	D67	<0.0001	0	51
5	K219	<0.0001	4	45
6	V118	<0.0001	2	39
7	L74	0.00034	0	29
8	K43	0.00062	0	28
9	L210	0.00099	6	40
10	K103	0.00233	0	24
11	T69	0.00324	2	29
12	K70	0.00361	0	23
13	L228	0.01271	0	21
14	K65	0.03923	0	17
15	Y181	0.03923	0	17
16	G190	0.03923	0	17
17	A400	0.04219	49	78

a Correction based on the False Discovery Rate (FDR) approach.

b p-values <0.05 are considered significant.

**Table 2:** Association between position 400 (alanine vs threonine) and polymerase domain mutations in subtype B viruses from patients failing therapy.

Mutated position	p-value*	A400 (%)	T400 (%)
M184	0.0004	30	65
T215	0.0036	28	57
L74	0.0037	5	27
V118	0.0045	10	34
K70	0.0063	3	22
D67	0.0086	18	42
K103	0.0112	3	19
K219	0.0229	18	39
M41	0.0310	25	46
Y181	0.0357	3	17
G190	0.0357	3	17

* p-values <0.05 are considered significant.

### Drug susceptibility of viruses containing substitution A400T

**Figure 1:**
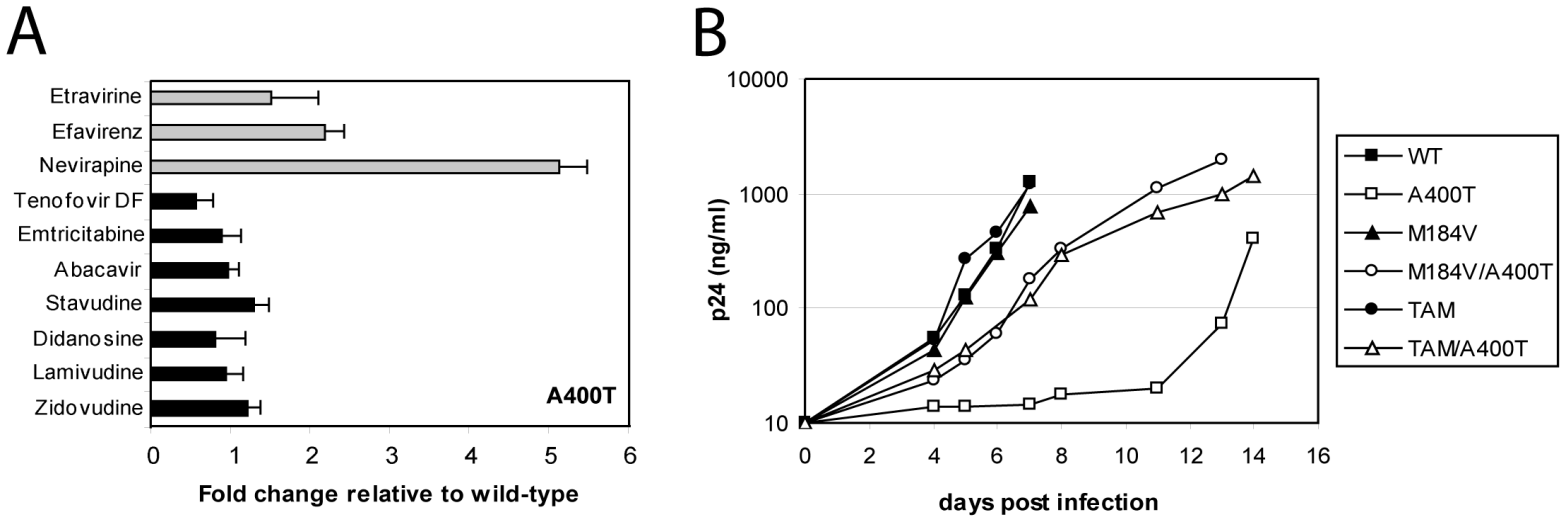
Drug susceptibility and replication of A400T viruses. (A) Susceptibility of the A400T virus to NNRTI (light grey) and NRTI (dark grey) was measured using single-cycle drug susceptibility assays. The average of three independent experiments is plotted, and the standard deviation is shown (error bars). The susceptibility of the A400T virus is shown as the relative difference in IC_50_ compared to the wild-type pNL4.3-XN virus. (B) Replication of viruses containing A400T alone, or in combination with M184V or TAMs. SupT1 cells were infected with an equal amount of virus (50 ng). CA-p24 production was measured in the culture medium at several days post infection. A representative experiment is shown. Three independent experiments were performed with similar results.

To analyze the role of T400 in the connection subdomain of HIV-1 RT in drug the resistance, we substituted the alanine at position 400 by threonine in the HIV-1 molecular clone pNL4.3-XN by site-directed mutagenesis. Given that we observed a strong association between T400 and polymerase mutations M184V, mutation T215Y and several other TAMs, we also introduced the A400T substitution into molecular clones containing the M184V mutation or TAMs M41L and T215Y. Single-cycle drug susceptibility assays showed that the A400T substitution reduced susceptibility to NVP and EFV approximately 5-fold and 2-fold, respectively (Fig 1A). The susceptibility to the second generation NNRTI etravirine was not significantly changed. These effects on susceptibility were measured in the wild-type background, and independent of the presence M184V or TAMs (results not shown). These results show that T400 alone confers low-level resistance to these NNRTIs. However, T400 by itself did not affect the sensitivity to NNRTIs (Fig 1A), neither did it combined with the M184V or TAM mutations (results not shown).

### Replication capacity of A400T viruses

To study the replication potential of the viruses containing T400, we used equal amounts of virus to infect SupT1 cells (Fig. 1B). These cells express the CD4-CXCR4 receptors, and are fully susceptible for replication of the pNL4.3 strain. Virus production was followed by measuring CA-p24 levels in the culture medium at several days after infection. The replication of the A400T virus was reduced compared to the wild-type virus, T400 also reduced the replication capacity of the M184V and TAM viruses. Similar results were obtained upon direct transfection of the molecular clones into the SupT1 cell line (results not shown). These results show that a fitness cost is associated with replacing the alanine at position 400 with a threonine in the HIV-1 NL4.3 virus strain.

### Effect of A400T on polymerase activity

**Figure 2:**
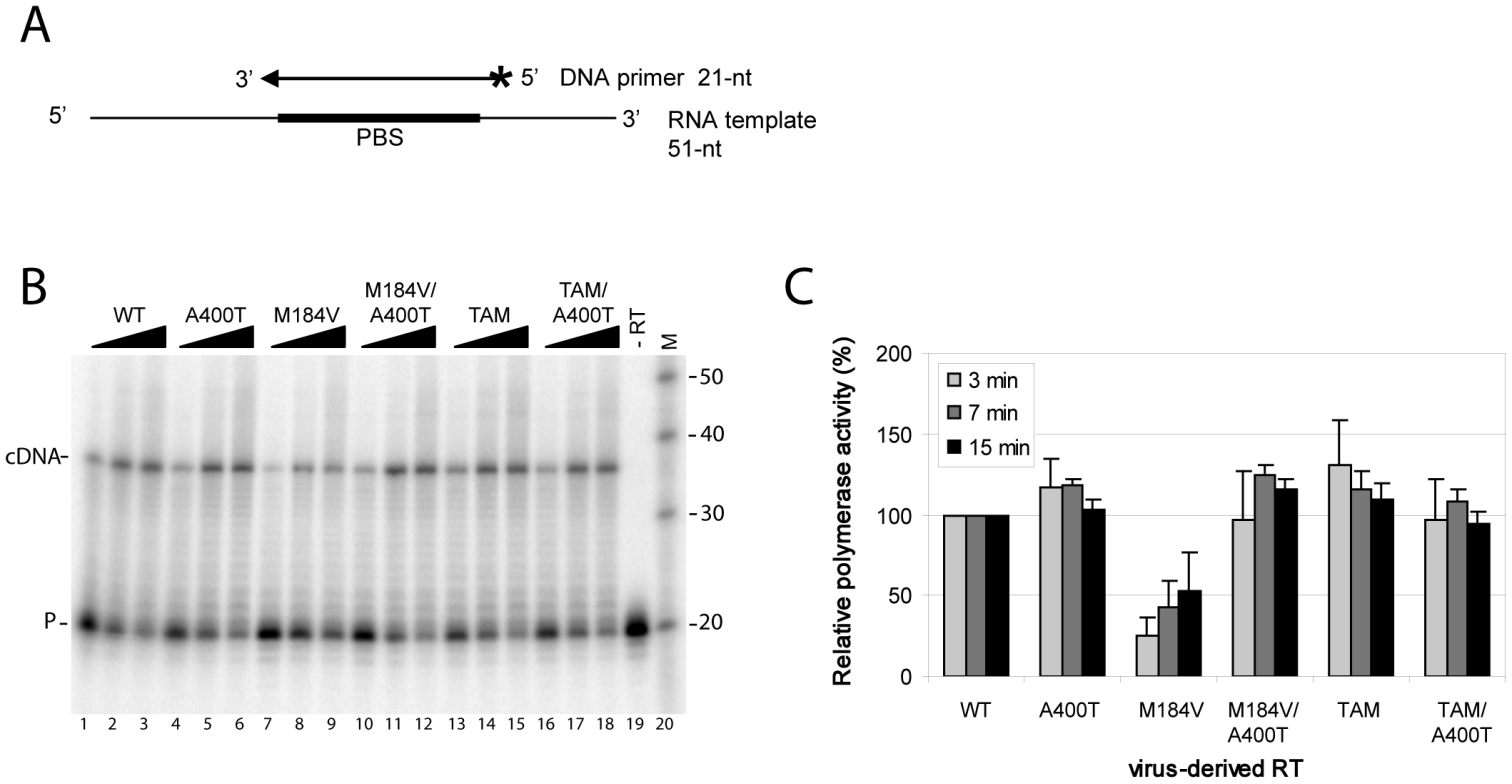
DNA polymerase activity of virus-derived RT enzymes containing substitution A400T. (A) DNA polymerase activity was measured using a 51-nt RNA template, representing the HIV-1 primer binding site (PBS) with flanking sequences. A 21-nt DNA primer complementary to the PBS was 5′-end-labeled (asterisk), and heat-annealed onto the RNA template. Extension of the primer by RT will result in a 38-nt labeled cDNA product. (B) The RNA template/DNA primer complex was incubated with the different virion-derived RTs in the presence of dNTPs. Formation of the cDNA product (cDNA) was monitored after 3, 7 and 15 min incubation. A control reaction was performed in the absence of RT (lane 19) showing only the labeled primer (P). (C) The amount of cDNA production was quantified in three independent experiments. Plotted is the average cDNA production with standard deviation (error bars). The polymerase activity of the wild-type virus-derived RT was set at 100%.

To characterize the effects of T400 on polymerase function, the activity of the virion-derived RT enzymes was analyzed (Fig. 2). Therefore, viral lysates were generated and normalized for the amounts of CA-p24. We used an RNA template encompassing the HIV-1 primer binding site (PBS) region and a labeled DNA primer complementary to the PBS (Fig 2A). Minus-strand DNA synthesis was initiated by the addition of virion-derived RT and dNTPs. Extension of the 21-nt primer results in increasing amounts of the 38-nt labelled cDNA product over time for the wild-type RT (Fig 2B, lanes 1–3). The amounts of cDNA products generated by the different virion-derived RTs were quantified, and the results of three independent experiments are plotted in Fig. 2C. Substitution A400T did not significantly affect the polymerase activity of the wild-type or TAM enzymes. Interestingly, the polymerase activity of M184V was reduced compared to the wild-type RT, and introduction of a threonine at position 400 restored this defect.

### Effect of A400T on RNaseH activity

**Figure 3:**
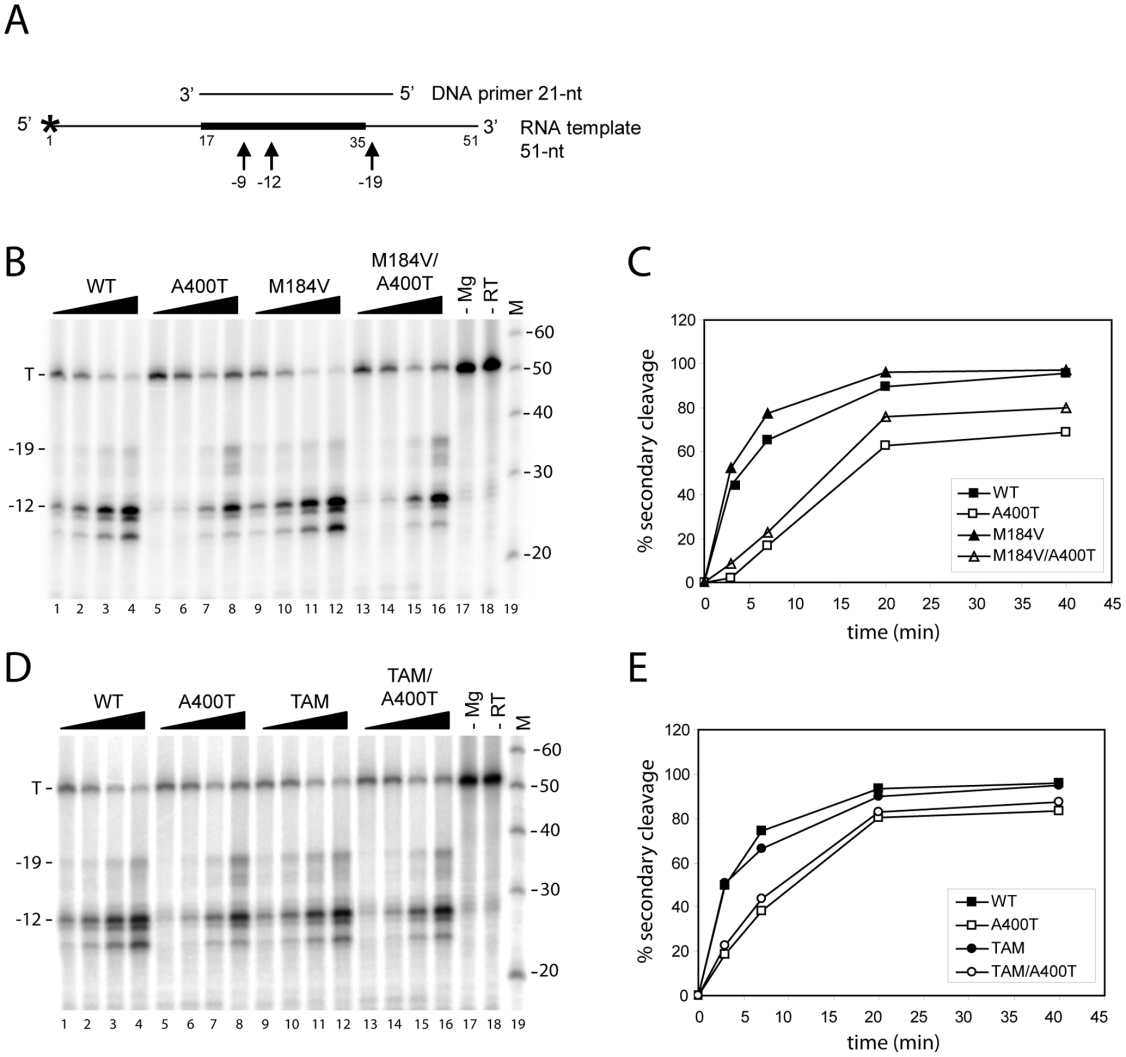
RNaseH activity of virus-derived RT enzymes containing substitution A400T. (A) RNaseH cleavage was measured using a 5′-end labeled (asterisk) 51-nt HIV-1 RNA template representing the PBS region. A 21-nt DNA primer complementary to the PBS was heat-annealed onto the RNA template. Primary (−19) and secondary (−12/−9) cleavages were monitored. (B) Representative gel showing RNaseH cleavage mediated by the virion-derived RTs with substitution A400T in the wild-type and M184V background. The reactions were initiated by the addition of Mg^2+^, and template (T) cleavage was monitored after 3, 7, 20 and 40 min of incubation. Control reactions were performed in the absence of Mg^2+^ (lane 18) or RT (lane 19), showing no template degradation. (C) The formation of secondary cleavage products over time was quantified, and is plotted for this representative gel. (D) RNaseH cleavage mediated by the virion-derived RTs with substitution A400T in the wild-type and TAM background was analyzed, and formation of secondary cleavage products is plotted in (E). Representative experiments are shown, and three independent experiments were performed with similar results.

A threonine at position 400 may influence the RNaseH activity of the RT enzyme, as was reported for other connection subdomain mutations [34]. We therefore investigated the RNaseH activity of the virus-derived RTs using a 5′-end-labeled RNA template/DNA primer as a substrate (Fig 3A). RNaseH cleavage was monitored over time, showing the formation of shorter RNA products due to primary (position −19) and secondary cleavages (positions −12 to −9) for the wild-type RT (Fig 3B, lanes 1–4). RNaseH cleavage was found to depend on the presence of magnesium (lane 17), and no degradation was observed in the absence of RT (lane 18). Formation of secondary cleavage products was quantified for the different virion-derived RTs (Fig 3 B/D). Mutation M184V did not affect RNaseH cleavage of the RNA template compared with the wild-type RT (Fig 3 B/C). However, substitution A400T reduces RNaseH activity in both the wild-type and M184V genetic backgrounds. Furthermore, TAM mutations did not affect RNaseH cleavage compared with the wild-type RT, whereas T400 in this background reduces RNaseH activity (Fig 3 D/E). These results indicate that threonine at position 400 reduces RNaseH activity both on its own and when combined with M184V or TAMs.

### Conformational changes in RT mediated by A400T

**Figure 4:**
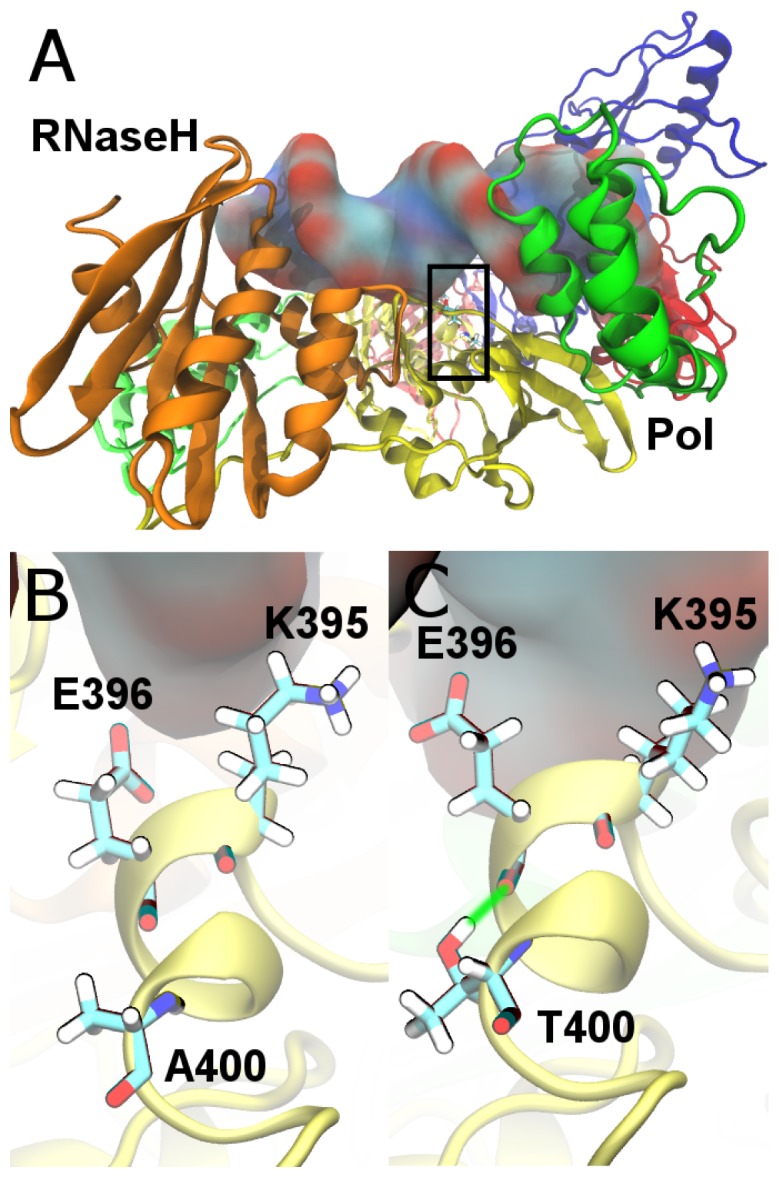
Structure of the HIV-1 reverse transcriptase (RT) enzyme. (A) Shows RT bound to a DNA/DNA template/primer complex (shown in surface representation). The enzyme is shown in cartoon representation and coloured by subdomain; fingers are blue, palm red, thumb green, connection yellow and the RNaseH orange. The p66 subunit is shown in the foreground and in darker shades. A black rectangle indicates the location of the p51 RNaseH primer grip. (B) and (C) show detailed views of this region, including chemical representations of the residues K395 and E396 in the wild-type and A400T RT enzymes, respectively. A green line is used to illustrate the hydrogen bond formed between T400 and E396.

Residue 400 is located distant from both the NNRTI binding pocket and the RNaseH active site, but was found to impact both drug susceptibility and enzymatic function. To investigate the structural changes in RT mediated by the alanine to threonine change at position 400, we performed molecular dynamics simulations. In the p51 subunit, residue 400 is located close to two residues involved in the RNaseH primer grip, K395 and E396 (all three residues are found in the alpha helix conventionally labeled αL [2] (Fig 4). The RNaseH primer grip has been shown to help position the template/primer complex at the RNaseH active site [56], [57]. In p66 these residues are located in a solvent exposed helix far from the functionally significant regions of the protein (see Fig S1 in File S1). We have previously demonstrated that the region around αL in p51 can be impacted by the binding of NVP [33]. The aim of our simulations is to test whether the change at position 400 alters the dynamics of residues in the RNaseH primer grip and whether any such changes are correlated with alterations in the template/primer trajectory or conformation. Simulations were performed on the NL4.3 wild-type and A400T HIV-1 RT enzyme bound to DNA/DNA and RNA/DNA template/primer complexes. These simulations were also performed for the RT enzyme bound to NVP. A 20 ns simulation trajectory was generated for the different RT complexes. No significant differences in the overall structure and flexibility of the polymerase domain or conformation of the RNaseH domain were observed between the wild-type and A400T enzymes for any of the simulated complexes, apart from in the highly flexible p66 fingers subdomain (see Fig S4–S7 in File S1). However, changes were observed in the region surrounding residue 400 in p51, which was therefore analyzed in more detail.

**Figure 5:**
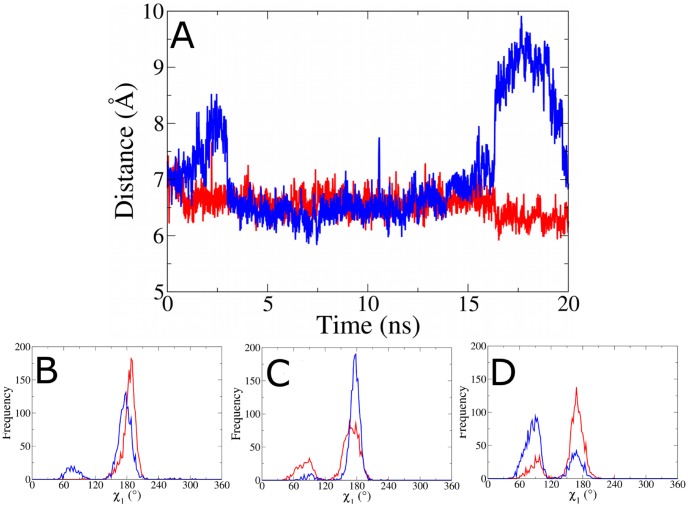
Distance between the scissile phosphate (−19) and the centre of mass of the RNaseH active site (p66 residues 443, 478, 498 and 549) over the course of the simulations of the HIV-1 RT bound to an RNA/DNA substrate. The simulation for the wild-type A400 is shown in red, and for substitution T400 in blue. Frequency distributions of the χ_1_ dihedral of p51 residue E396 in simulations of (B) RNA/DNA hybrid substrate, (C) double stranded DNA substrate and (D) NVP bound HIV-1 RT (results for the unbound RT are shown in Fig S16 in File S1). The simulation for A400 is shown in red, T400 in blue.

In all simulations with a threonine at position 400 a stable hydrogen bond is formed between the side chain oxygen of T400 and E396 (Fig 4B/C). We propose that formation of this hydrogen bond results in subtle changes in the backbone of the αL helix. To investigate how these affect the RNaseH primer grip we measured the side chain dihedral angles of p51 residues K395 and E396 (Fig S8–S10 in File S1). No significant changes were observed in the χ_1_ dihedral of residue 395. Differences are however evident in the χ_1_ dihedral angle of E396. The flexibility of the sidechain in the RNA/DNA-bound A400T RT enzyme was greater than that in the wild-type RT complex (Fig 5A and Figure S9 in File S1). In wild-type RT only angles close to 190° are found, whereas in A400T the distribution of angles is bimodal with peaks around 180° and 80°. Interestingly, the opposite change in flexibility was observed for the RT enzymes bound to the DNA/DNA complex. Only a single peak is found in the χ_1_ angle distribution for the A400T enzyme, and a bimodal distribution is observed for the wild-type (Fig 5B). As we have previously shown [33], this region is also impacted by the binding of NVP with both RT enzymes showing bimodal distributions with peaks around 90° and 170° (Fig 5C). In wild-type RT, the 170° angle is more frequently observed, whereas the 90° angle is more frequent for A400T. This suggests that a threonine at position 400 would be able to oppose the effects of NVP binding to the DNA/DNA template/primer RT complex.

The differences observed in E396 change the pattern of salt bridging between this residue and p66 residues 356 and 358. In the 2HMI crystal structure (used as the basis for the DNA/DNA simulations) a salt bridge is formed between residues 396 and 358. During the simulations of the DNA-bound RT two conformations are sampled, one which maintains the salt bridge with residue 358 with an average length of 2.81 Å, and another in which a salt bridge forms with residue 356 with an average length 2.90 Å. These residues are in a loop that links directly to residues within the RNaseH primer grip. We observe that these small changes in residue flexibility are accompanied by larger scale changes in the orientation of p51 K395 and E396 relative to p66 primer grip residues 356 and 358 in the RNaseH domain. The angle between the p51 and p66 residues was affected by template/primer- and NVP-binding for both the wild-type and A400T RT (Table 3 and Fig S11 in File S1). The largest change was measured with the A400T RT upon binding of the DNA/DNA complex. The angle was reduced to 64° for A400T, whereas an angle 110° of was measured for the wild-type RT. The orientation of the primer grip residues was less affected in the A400T RT by binding of the RNA/DNA complex or NVP compared to the wild-type. This suggests that the RNA template may not be correctly aligned for degradation when a threonine is present at position 400 in RT and this effect may be enhanced by NVP binding.

**Table 3:** The average angle between RNaseH primer grip residues in the p51 (residues 395 and 396) and p66 (359 and 360) subunits observed in simulations of HIV-1 RT with alanine and threonine at position 400.

	A400	T400
Unbound	130° (8)	121° (11)
DNA/DNA	102° (6)	64° (7)
RNA/DNA	110° (7)	120° (8)
NVP	94° (9)	103° (11)

Standard deviations are shown in parentheses.

We then investigated if T400 in the RNaseH primer grip changes the template/primer trajectory. The distance between the scissile phosphate (−19) and the centre of mass of the RNaseH active site (p66 residues 443, 478, 498 and 549) was analyzed over the course of the simulations (Fig 5D). We detected a constant distance of around 6.5 Å for the wild-type RT. This distance is more variable in the A400T RT, for which values between 6 and 9.5 Å were observed. These changes in the trajectory of the template/primer suggest that the A400T RT is less capable of maintaining the catalytic conformation. The incorrect orientation of the scissile phosphate relative to the catalytic residues may result in reduced RNaseH cleavage. Alongside the alteration of the template trajectory and conformation we observe changes in the flexibility of the template. The RNA template was found to be more flexible than the DNA template in the primer/template-bound RT simulations (S12–S13), which is in agreement with experimental evidence [58]. It has been suggested that these differences are responsible for substrate recognition by RNaseH. Particularly flexible is the region between base pairs −10 and −12, which corresponds with the location of p51 residues 395 and 396 in the RNaseH primer grip.

It has been suggested that recognition by HIV-1 RT of template/primer complexes, and their alignment by the primer grip [56], is influenced by the width of the substrate minor groove [59], [60]. The RNaseH primer grip residues directly interact with the minor groove and alterations in its width and flexibility are observed between alanine and threonine at position 400 in our simulations (Fig S12–S15 in File S1). The largest changes were observed between bases −12 and −14 of the DNA/DNA complex bound to the A400T RT enzyme, although these may be exaggerated by the short length of the simulated duplex. These effects are in agreement with the detected change in the primer grip angle (Table 3). For both template/primer complexes the changes in flexibility and minor groove width are only local to the RNaseH (and primer grip) domain, and do not affect the conformation at the polymerase active site. These combined results suggest that T400 affects the conformation of the RNaseH primer grip, and changes the position of the RNA template relative to the scissile phosphate in the RNaseH active site. This conformational change is likely to reduce the catalytic rate and explains the reduced RNaseH cleavage that was measured for A400T RT in biochemical assays (Fig 3).

We also investigated the structural effects of position 400 in other HIV-1 subtype B isolates. The HXB2 isolate contains a threonine at position 400, and crystal structures indicate that this forms the same hydrogen bond with E396. However, several residues near position 400 differ between the NL4.3 and HXB2 viruses. For instance, the p66 residue 376 is located in close proximity to the αL helix that contains residue 400 in p51 (see Fig S17 in File S1). The NL4.3 virus contains hydrophobic alanine residues at positions 400 and 376, whereas HXB2 contains polar threonine residues. The favourable interactions between positions 376 and 400 allow close packing at the dimer interface. This is disrupted by substituting alanine for threonine in NL4.3, resulting in distortion of the local geometry. We noticed that the residues at positions 376 and 400 co-vary in different HIV-1 subtype B isolates. In the Los Alamos HIV database, 37% of the sequences contain A400, whereas 52% contain T400. Analysis of the sequences with A400 shows that 81% has an alanine, and only 17% has polar threonine or serine residues at position 376. In contrast, when T400 is present, the frequency of T/S376 is increased to 43%, whereas A376 is reduced to 54%. This co-variation is highly significant (p<0.0001), however other residues are likely to play a role as the T400/A376 combination is found in approximately half of the subtype B sequences. Local interactions in the RT protein thus may determine the selection of alanine or threonine at position 400 in HIV-1 subtype B viruses.

## Discussion

Mutations in the connection and RNaseH domains of HIV-1 RT were previously found to confer resistance to NRTI and NNRTI [26], [27]. In this study, we identified a novel role for polymorphism T400 in the connection domain of RT. We analyzed the complete RT sequences of 135 naïve and treated patients infected with subtype B HIV-1 in France. Position A/T400 is polymorphic in naive individuals, but T400 occurs more frequently in patients failing therapy. No other statistically significant mutations in the connection or RNaseH domain were found to be associated with treatment failure. Several positions involved in NRTI and NNRTI resistance were found to be associated with T400, in particular M184V/I and T215Y/F. To study the role of position 400 in drug resistance, the alanine at position 400 in the reference strain NL4.3 was substituted by a threonine using site-directed mutagenesis. Substitution A400T was also introduced in combination with M184V and TAMs T215Y and M41L. Drug susceptibility assays demonstrated that T400 mediates low-level resistance to NVP (5-fold IC_50_ increase) and EFV (2-fold) in the wild-type NL4.3 background. However, T400 alone, or in combination with M184V or TAMs, did not affect the sensitivity for NRTIs. An increase in proportion of T400 was found to be associated with exposure to NVP in treated HIV-1 patients, which is consistent with the measured decrease in NVP susceptibility. In addition, an association was found with exposure to AZT and ABC, but no change in susceptibility to these NRTIs was measured.

Analysis of the RT sequences showed a 41% prevalence of T400 in untreated patients, which increased to 72% in patients failing therapy. A previous study also reported an increase in the frequency of T400 from 43% in naïve to 69% in treated subtype B patients in Brazil [28], [29]. We analyzed the prevalence of T400 in two major HIV sequence databases. The Los Alamos HIV database collects published HIV sequences that were not generated during drug resistance studies, whereas the Stanford HIV drug resistance database accepts sequences only from patients developing virological failure. The prevalence of T400 varies between 52% in the Los Alamos database to 41% in the Stanford database for untreated HIV-1 subtype B patients. The increase in the frequency of T400 in treated patients is not found in the Stanford database. Analysis of the database at Janssen Diagnostics in which the results of 446007 Antivirogram phenotypic assays are collected [61], showed a 76% prevalence of T400, combined with reduced susceptibility to the NNRTIs NVP, EFV and ETR. Differences in the time and place of infection, risk group or route of infection of the patient sequences collected in France, Brazil and the databases may have resulted in differences in the prevalence of T400. Differences in treatment regime or time of sampling after failure may account for variation in the percentage of T400 found after treatment. Furthermore, viruses with classical NNRTI resistance mutations (such as K103N, Y181C) in the polymerase domain may eventually replace the viruses with T400 due to much higher resistance levels conferred by these mutations.

Previous studies that analyzed HIV-1 subtype AE and C sequences also showed an increased prevalence of polymorphism T400 after treatment. In subtype AE, T400 was found to be associated with NRTI treatment [30], [31]. In the presence of four TAM mutations (D67N, K70R, T215Y and K219Q), residue T400 was found reduce the susceptibility to AZT by increasing its excision [31]. In subtype C, T400 mediated a two-fold increase in AZT resistance when combined with four TAM mutations [32]. However, in our study in the HIV-1 subtype B NL4.3 background, T400 did not further increase resistance to AZT mediated by the two TAM mutations M41L and T215Y. These results suggest that the background, and the number and specific (combinations of) TAM mutations determine the levels of AZT resistance conferred by polymorphism T400.

This is the first study to describe resistance to NNRTIs mediated by position T400 in RT. Susceptibility to NVP was reduced 5-fold, whereas that of EFV was reduced 2-fold in the wild-type NL4.3 background. A number of other amino acid substitutions in the RT connection subdomain have been associated with low-level (<2-fold) resistance to NNRTIs [27]. In some cases, the residues involved occur at the NNRTI binding pocket and participate in interactions with the inhibitor (e.g. Y318F/W) [62], [63]. But most drug resistance mutations occur away from the NNRTI binding pocket. The molecular mechanisms involved in resistance mediated by these mutations are less clear and still being investigated. The most extensively studied mutation is N348I, which provides resistance to AZT and NVP [23], [64]–[68]. This mutation was shown to reduce the rate of RNA template degradation by RT, giving RT more time to excise the blocking nucleoside analogue from the terminated primer [23], [69]. A general model to explain dual resistance to NRTI and NNRTI based on RNaseH activity has been proposed [35]. Reduced RNaseH cleavage of the RNA template will allow more time for dissociation of the NRTI from the RT-template/primer complex, leading to more efficient re-initiation of DNA synthesis.

Consistent with this model, we measured reduced RNaseH activity due to T400 in the HIV-1 NL4.3 isolate either alone or when combined with M184V and TAMs. These results suggest that this substitution decreases sensitivity to NVP and EFV by reducing RNaseH cleavage of the RNA template, allowing more time for dissociation of the inhibitor and continuation of DNA synthesis. Interestingly no significant resistance to the second generation NNRTI ETR was measured. ETR represents a different class of inhibitors, that is able to bind the enzyme RT in multiple conformations and thereby escape the effects of drug-resistance mutations [70]. EFV is known as a tight binding inhibitor [71], whereas NVP dissociates more frequently from the RT-template/primer complex [72]. This may explain the higher resistance to NVP (5-fold) compared to EFV (2-fold).

We show that the A400T substitution did not affect the DNA polymerization activity of the wild-type and TAM RTs. However, a reduced polymerase activity was measured for the M184V mutant, as was previously reported [18]–[20]. Residue T400 was found to restore the polymerization defect mediated by M184V (Fig 2). The process of reverse transcription depends on a delicate balance between DNA synthesis and RNA degradation. Substitution A400T reduces RNA template degradation, which may provide more time for the less processive M184V mutant RT enzyme to reverse transcribe the RNA template. Similar results were obtained for mutation N348I, which was also shown to compensate for mutation M184V [73]. Substitution A400T was found to reduce the virus replication capacity in the absence of inhibitors, also in the M184V background. Thus, although T400 was able to restore the polymerization defect of M184V, the reduced RNaseH activity may affect other steps in the reverse transcription process, such as strand-transfer and primer-removal, resulting in reduced replication. These findings imply a fitness cost is associated with the alanine to threonine change at position 400 in the HIV-1 NL4.3 virus strain. Our structural analysis suggests that this is the result of distortion of the region surrounding position 400, as the polar threonine clashes with the hydrophobic alanine at position 376. However, in other HIV-1 isolates, as for example HXB2, T400 is combined with T376 which compensates for these structural changes. Substitution T376, and possibly other local interactions, thus may restore the replication defect mediated by T400, explaining the presence of the T400 in a proportion of the naïve HIV-1 subtype B patients. In addition, we showed that replication of the wild-type NL4.3 virus was more severely affected by the A400T substitution than replication of the M184V and TAM viruses. This indicates that polymerase mutations can partly compensate for the replication defect associated with T400.

It is curious that our data demonstrate a strong association of T400 with M184V/I despite it having no significant effects on drug susceptibility in the context of M184V, and no positive effects on viral fitness. One possible explanation for this may lie in the previously observed antagonism between M184V and TAMs. Mutation M184V suppresses the resistance conferred by TAMs, although this effect decreases with an increase in the number of TAMs [74]. It is possible that T400 might be involved in facilitating dual resistance to AZT and 3TC resistance in viruses that harbour both TAMs and M184V, as was suggested for mutations G333D/E and N348I in the connection subdomain [75], [76]. Further studies in which substitution A400T is introduced in a background with more TAM mutations in combination with M184V will be needed to address this possibility.

Using molecular dynamics simulations we provide a novel atomistic mechanism for the changes in the RNaseH activity induced by the alanine to threonine change at position 400 in the HIV-1 RT. The position is located far from both the polymerase and RNaseH active sites of the enzyme. Simulations showed no significant differences in the structure and flexibility of the polymerase domain, but changes were observed in the RNaseH domain. A hydrogen bond is formed in the p51 subunit between residue T400 and E396 which is part of the RNaseH primer grip (Fig 4). This hydrogen bond was also found in simulations of template/primer- and NVP-bound RT with a threonine at position 400. Simulations showed that the distance between the scissile phosphate (−19) and the RNaseH catalytic centre is more flexible for T400. This may result in the incorrect orientation of the scissile phosphate relative to the RNaseH catalytic residues. We also show that T400 changes the salt bridging in the primer grip region, thereby changing the orientation of several residues in the primer grip. The RNaseH primer grip residues directly interact with the minor groove of the RNA/DNA substrate, and changes were detected in the width of the minor groove. Our simulations suggest that substitution A400T changes the conformation of the RNaseH primer grip region. We have proposed a detailed mechanism in which these changes are initiated by a hydrogen bond between T400 and E396, however further structural studies are required to more completely describe the molecular basis of the effects we have observed. The structural changes in the RNaseH primer grip alter the trajectory of the viral RNA genome, thereby reducing the accessibility of the correct catalytic conformation. These changes likely decrease the RNaseH catalytic rate of the A400T RT, leading to reduced RNaseH cleavage. These results support the previous hypothesis that the T400 substitution in subtype AE may disrupt the interaction with E396, thereby altering the primer grip region and reducing RNase H activity [31]. The slower degradation of the viral RNA genome may provide more time for dissociation of the bound NNRTI from the stalled RT-template/primer complex. Consequently the RT enzyme can resume reverse transcription, explaining the observed resistance. NVP binding was observed to impact the conformation of the same region, further supporting the hypothesis that interactions between the RNaseH primer grip and NNRT binding pocket are responsible for the resistance mediated by the A400T substitution.

The extent to which connection and RNaseH domain mutations influence a patient's clinical outcome is yet to be determined. However, it is evident that several mutations in the connection and RNaseH domains have increased prevalence amongst treatment-experienced patients, and contribute to drug resistance. In this study, we demonstrated that a threonine at the polymorphic position 400 confers low-level but significant resistance to the NNRTIs NVP and EFV in the HIV-1 subtype B NL4.3 background. We found an association between T400 and resistance mutations M184V/I, TAMs and several positions involved in NNRTI resistance. Previous studies reported resistance to AZT mediated by T400 in subtypes AE and C combination with four TAM mutations [31], [32]. These results suggest that drug susceptibility of viruses containing T400 may depend on the number and specific (combinations of) mutations in the polymerase domain. Future studies will focus on the effects of T400 in combination with NNRTI resistance mutations, e.g. K103N or Y181C. Since T400 is highly associated with both M184V and TAM mutations, it will also be interesting to determine if T400 can counteract the antagonism between M184V and TAMs.

## Supporting Information

File S1 **Supplemental material.**
(PDF)
