# Supplementary material for: A Polymorphism at Position 400 in the Connection Subdomain of HIV-1 Reverse Transcriptase Affects Sensitivity to NNRTIs and RNaseH Activity
Source: PLoS One. 2013 Oct 2;8(10):e74078. doi: 10.1371/journal.pone.0074078 (PMC3788777; doi:10.1371/journal.pone.0074078)
Supplement: File S1 — Supplemental material. (PDF) [file pone.0074078.s001.pdf]

Supplemental Material

A polymorphism at position 400 in the connection subdomain of HIV-1 reverse transcriptase affects sensitivity to NNRTIs and RNaseH activity

Wright et al.

Structure creation and simulation protocol

We have simulated models of HIV-1 RT bound to NVP (PDB: 1VRT), double stranded DNA (PDB: 2HMI), a hybrid RNA/DNA duplex based on the polypurine tract (PDB: 1HYS) and the unbound enzyme (PDB: 1DLO). Unfortunately, in all available HIV-1 RT crystal structures some residues are not resolved and a number of loop residues in the p51 subunit of the NNRTI bound structures used here are missing in the PDBs. The models were completed by copying in the coordinates for the missing loop residues from PDB: 1HQU (this structure was chosen due to its high resolution, 2.7 Å, and completeness in regions missing from other structures) after alignment of the surrounding residues using VMD [1]. These structures were then used as templates to homology model the NL4.3 wild-type and A400T sequences using the Deepview package (formerly known as Swiss-PdbViewer) [2]. This involved the fitting of the common atoms of the residues in the target sequence to those in the edited PDB template. the following mutations were made to transform the HXB2 background sequence of the crystal structures into NL4.3: Q102K, K122E, K162S, F214L, A272P, K358R, A376T, I343V, D460N, P468T, H483Y, K512Q and S519N. In each case the final model contains 556 residues in the first (p66) chain and 427 in the second (p51) chain for a total of 983 residues. Each system was solvated using a cubic box of TIP3P water molecules [3] providing a buffer of at least 14 Å distance around the protein. The systems were neutralised by the addition of Cl^- ions for the unbound enzyme and NVP bound complexes and Na^+ ions for those containing nucleic acids. Simulations for each system produced 20 ns of production simulation with structures output for analysis every 10 ps.

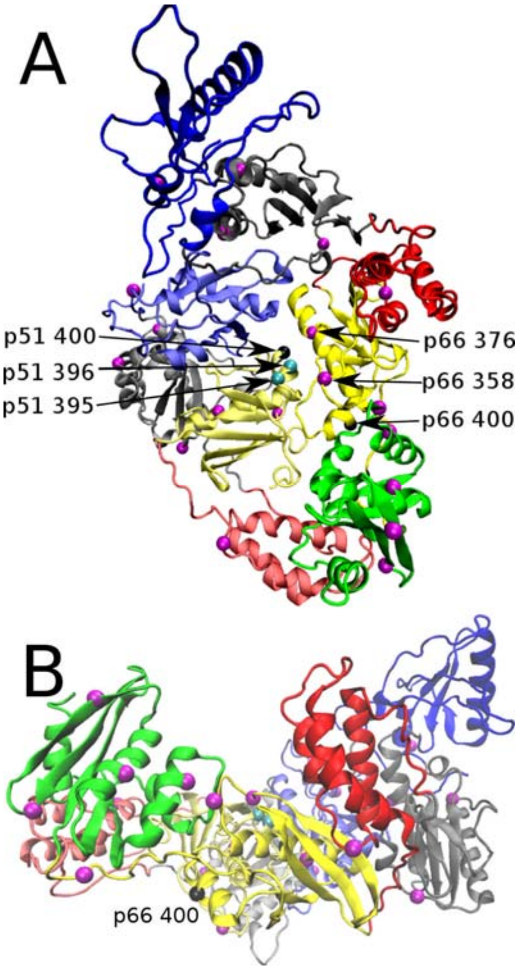

Fig S1: The positions in the HIV-1 RT sequence that differ between the NL4.3 and HXB2 are shown on the enzymes structure as magenta spheres, p51 residues 395 and 396 are shown as cyan spheres. (A) shows a view down into the template/primer binding cleft and (B) one from the side. The enzyme is shown in cartoon representation and coloured by subdomain; fingers are blue, palm red, thumb green, connection yellow and the RNaseH orange. The p66 subunit is shown in darker shades. Positions of significant residues in both subunits are labelled. Note that p66 residue 400 is located in a solvent exposed helix away from any functionally significant regions.

Inhibitor potential parameterization was performed by extracting the drug coordinates into separate files, using the PRODRG tool [4] to insert missing hydrogen atoms. The geometries were then optimized using Gaussian 98 [5] (with the 6-31G** basis functions). The Restrained Electrostatic Potential (RESP) procedure, part of the AMBER package [6], was used to calculate the partial charges. The force field parameters for the inhibitors were described using the General AMBER Force Field (GAFF) [7]. The protein and nucleic acid elements of all systems were described by the standard AMBER force field (ff03) [8] which is parameterized for bio-organic molecules including DNA in particular. The default variants (such as protonation states) for amino acids in physiological conditions were used for all residues.

The molecular dynamics package NAMD2 [9] was used throughout the minimisation, equilibration and production stages of the simulations. Electrostatic interactions were treated using the particle mesh Ewald (PME) [10] method and SHAKE [11] constraints were applied to all bonds involving hydrogen atoms in order to employ a 2 fs integration time step. Minimisation was conducted using the conjugate gradient and line search algorithms for 2000 iterations of each system. During this process all heavy atoms were restrained using a force constant of 5 kcal mol^{-1} Å^{2}. The next stage of the equilibration process was a mutational relaxation protocol in which each mutated residue and residues within 5 Å are released in turn from the restraints for 50 ps. This allowed the residues to re-orientate into more favourable conformations if necessary. After the 50 ps relaxation period the restraints are reapplied to each region. The equilibration phase anneals the system taking the temperature from 50 K to 300 K in 50 ps. Once achieved, the final temperature was maintained using a Langevin thermostat with a coupling coefficient of 5 ps^{-1}. This was followed by completely isothermal equilibration for 200 ps in the canonical (NVT) ensemble. In both of these stages the restraints imposed during minimization were retained. The restraints were then gradually reduced in four steps of 1 kcal mol^{-1} Å^{2}, each step running for 50 ps. The restraints applied are weaker than in the protease case as no regions are known to suffer solvation induced deformities unlike the flap region of the protease. After this, the restraints were removed completely and the systems allowed to evolve under isothermal-isobaric (NPT) conditions using a Berendsen barostat [12] with a target pressure of 1 bar and a pressure coupling constant of 0.1 ps. Coordinate trajectories were recorded every 1 ps throughout all equilibration and production runs. The five systems were built using extensions to the Binding Affinity Calculator (BAC) scripts created to automate simulations and free energy calculations for the HIV-1 protease [13]. The Application Hosting Environment (AHE) [14] was used in order to automate the running of simulations and retrieval of data.

Physical properties can only be reliably calculated from systems which have been adjudged properly equilibrated. For all systems simulated here the potential energy minimisation applied is sufficient to remove all bad contacts as measured by the decrease in potential energy which, after the heating phase, remains stable with a standard deviation of less than 450 kcal mol^{-1} in all cases. A further test of whether the systems under study have equilibrated was made by investigating the structural variation seen over the simulation. Using difference distance matrices, [15] determined a set of residues which vary in relative position by less than 2 Å in a wide range of HIV-1 RT crystal structures. These residues are assumed to represent the most structurally stable regions of the protein (listed in Tab S1). The root mean squared fluctuations (RMSF) of each system was calculated relative to the

average structure for these residues (HIV-1 RT is known to be a flexible protein with a number of loop regions for which conformational changes might be expected throughout even equilibrated simulations). The RMSF of the simulations reduced to, and remained below, 1.5 Å after 6 ns for all systems (See Fig S2). The root mean squared deviations for the protein backbone is also seen to plateau after around 5 ns (see Fig S3) further indicating that the systems have reached an equilibrated state following minimization and constraint removal. Consequently the trajectory between the start of the simulation and 6 ns in is defined as being the equilibration phase and all subsequent parts of the simulation comprise the production phase. All analyses presented here are derived from production phase simulations.

Tab S1: Residues determined to vary relative positions by less than 2 Å in a survey of HIV-1 RT crystal structures by Keller et al. [15].

**Chain**	**Residues**
p66	4-6, 95-107, 162-163, 180-181, 188-200, 202-205, 226, 234-235, 237-239, 317, 319, 323, 339-345, 349-353, 365-366, 368-402, 405-419, 428-436, 439, 493, 530
p51	6-7, 18-45, 54-64 71-84, 97-111, 113-117, 121, 123-138, 140-174, 176-184, 186-192, 197-198, 201-202, 208, 252, 254-264, 267, 274, 277, 280-282, 284, 296, 298-300, 303-307, 320, 322, 329, 331, 333-335, 364-393, 397-417

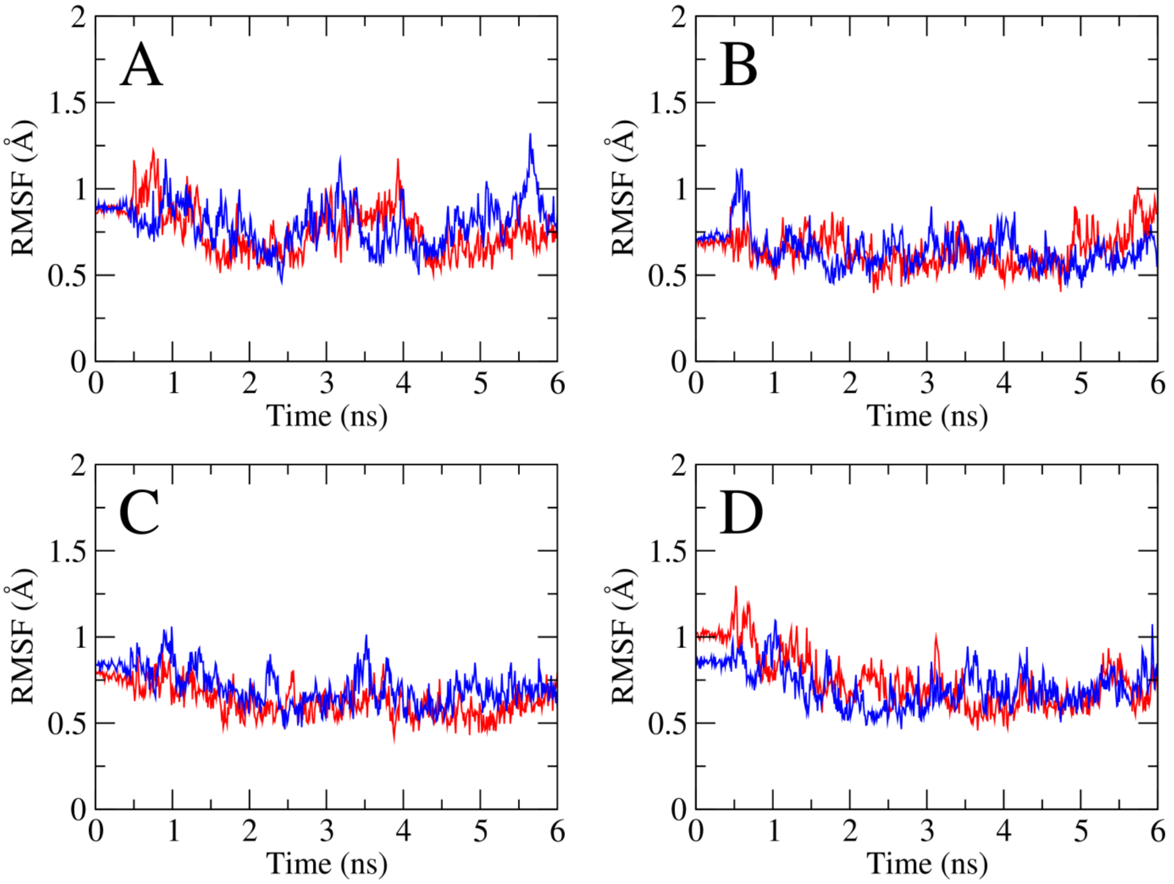

Fig S2: The RMSF of the residues of HIV-1 RT defined in Tab S1 as having minimal variation between cristal structures for (A) the unbound enzyme, and bound to (B) NVP, (C) RNA/DNA template/primer complex and (D) a dsDNA template/primer duplex. Red lines indicate the simulation of the wild-type NL4.3 RT sequence and blue the A400T RT sequence.

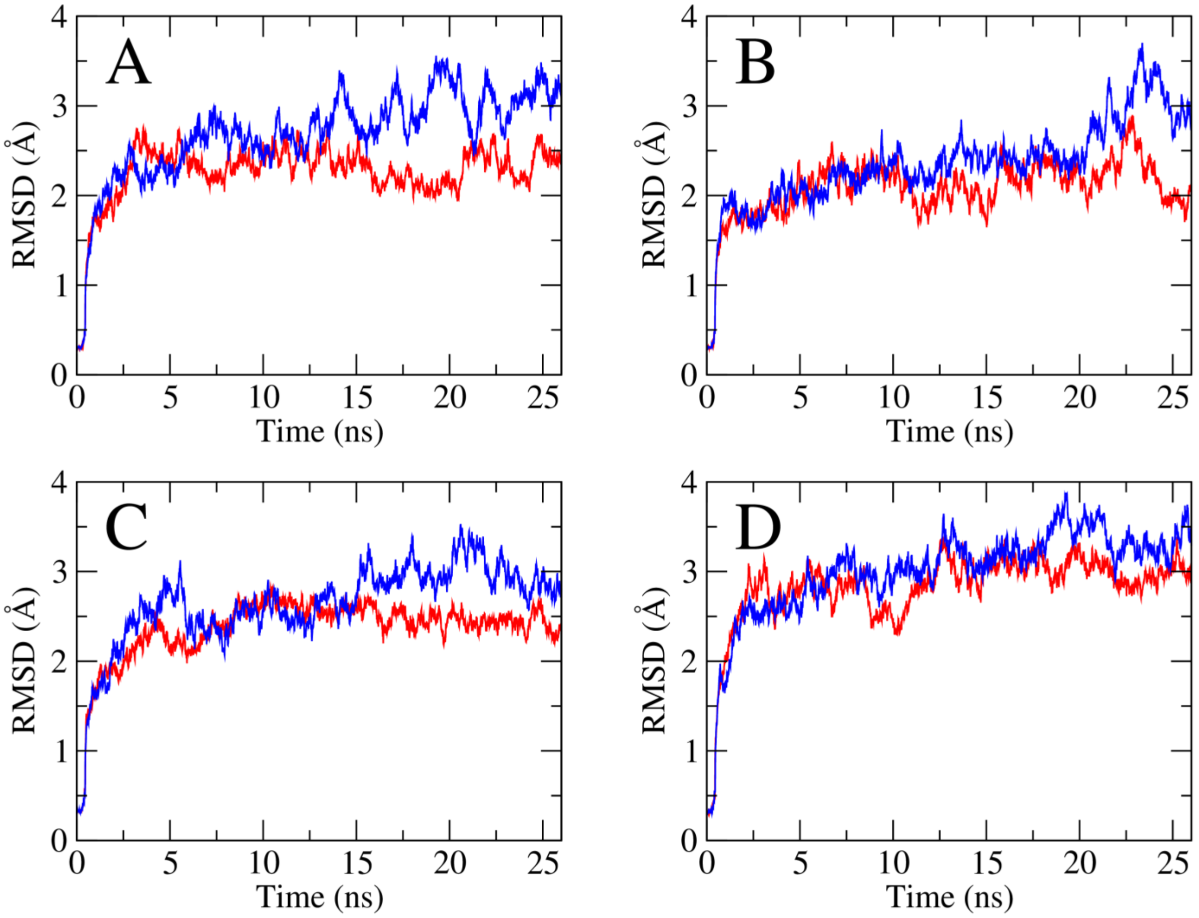

Fig S3: The RMSD of HIV-1 RT backbone over the couse of the simulations for (A) the unbound enzyme, and bound to (B) NVP, (C) RNA/DNA template/primer complex and (D) a dsDNA template/primer duplex. Red lines indicate the wild-type RT and blue the A400T RT.

Structure flexibility

The structural changes after equilibration are mainly confined to loop regions with only minor differences between the two sequences observable (see Figs S4-S7). The main differences are concentrated in the p66 fingers which are largely free to move in the absence of an incoming nucleotide or NRTI.

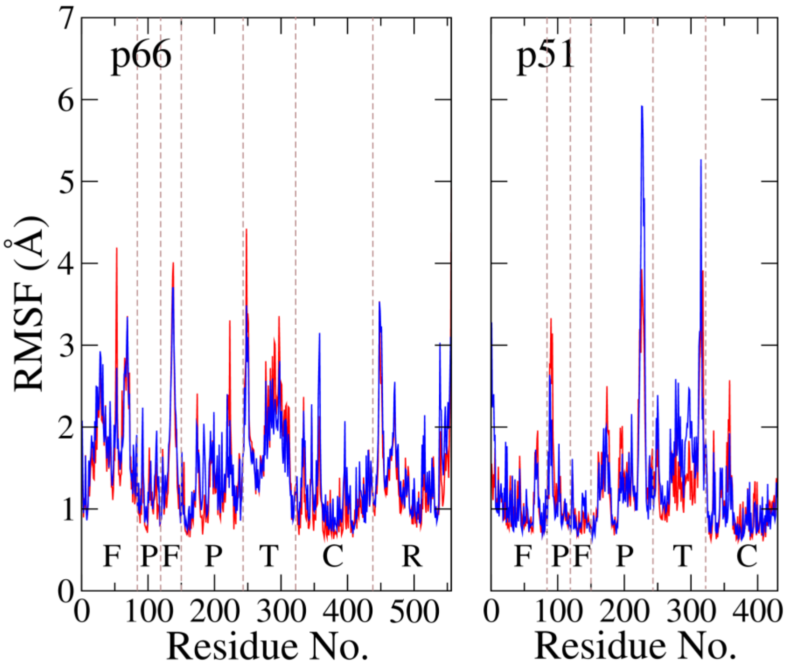

Fig S4: RMSF per residue of the unbound HIV-1 RT during the 20 ns of production simulation. Red lines indicate the wild-type RT and blue the A400T RT.

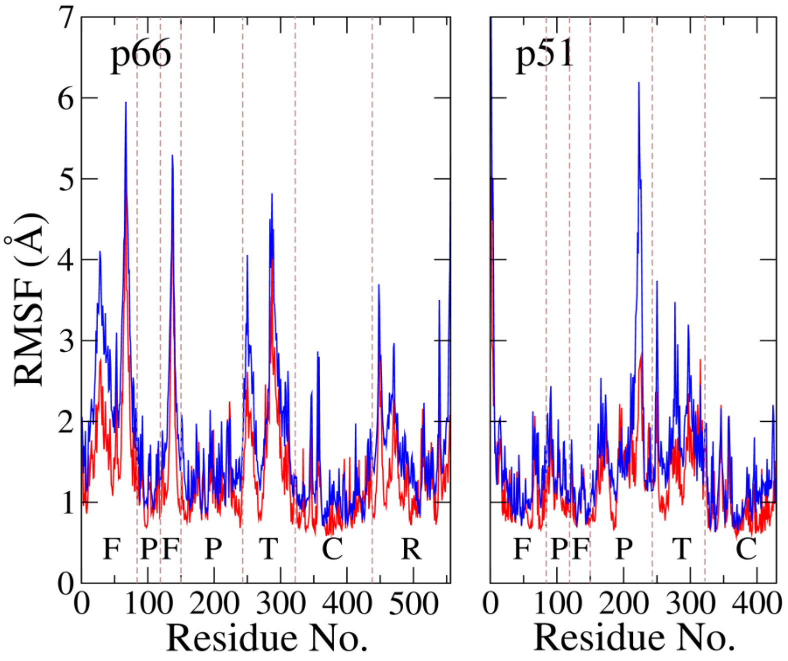

Fig S5: RMSF per residue of the NVP bound HIV-1 RT during the 20 ns of production simulation. Red lines indicate the wild-type RT and blue the A400T RT.

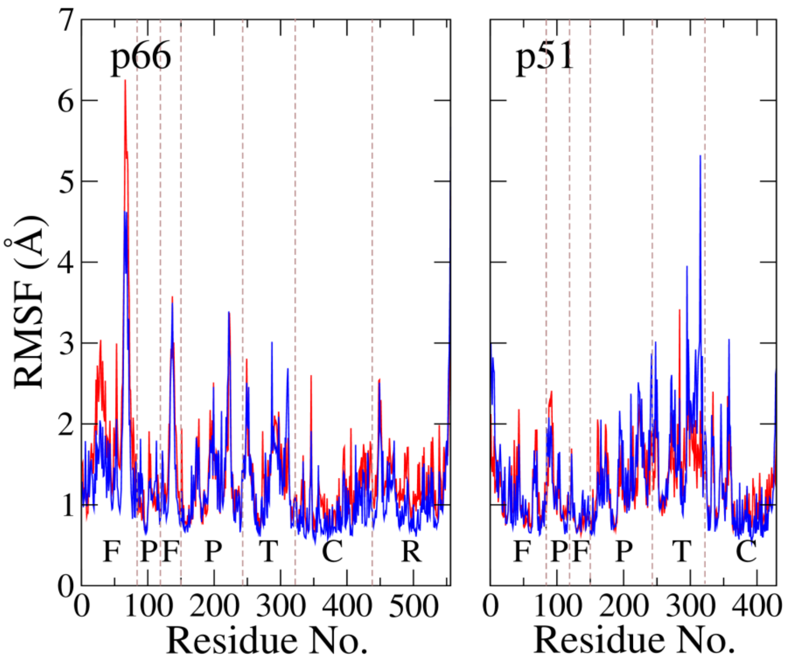

Fig S6: RMSF per residue of the HIV-1 RT bound to RNA/DNA template/primer complex during the 20 ns of production simulation. Red lines indicate the wild-type RT and blue the A400T RT.

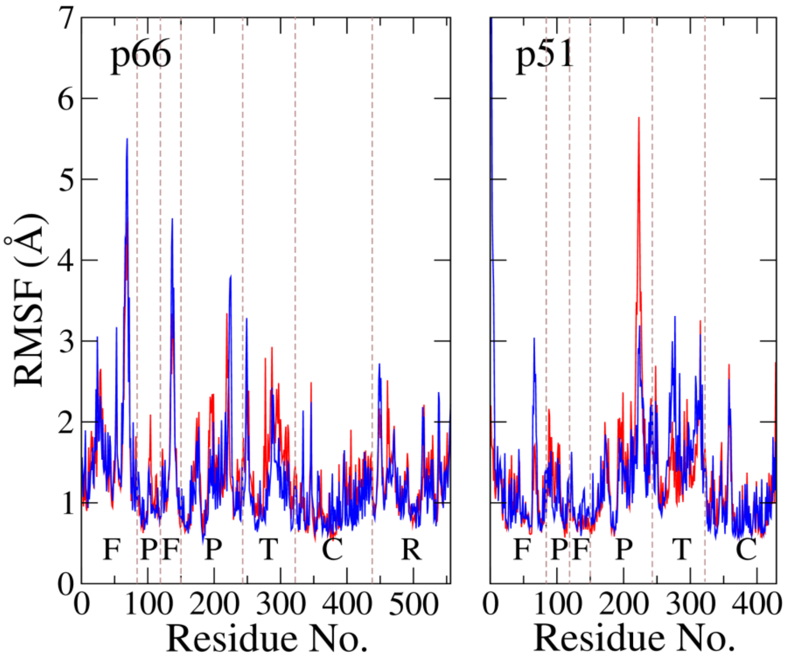

Fig S7: RMSF per residue of the HIV-1 RT bound to dsDNA template/primer during the 20 ns of production simulation. Red lines indicate the wild-type RT and blue the A400T RT.

Definition of dihedral angles

The dihedral angle φ defines the rotation of the fourth atom in a chain about the middle bond of three linking the atoms (see Fig S8). Changes in this angle only alter the distance between the first and fourth atoms (the other distances are defined by the chemical bonds between them). In amino acid side chains the successive sidechain dihedral angles are labeled as χ_1-χ_5 (depending on sidechain length). The χ_1 dihedral angle is defined by atoms N-C_α-C_β-C_γ, the χ_2 dihedral by atoms C_α-C_β-C_γ-C_δ.

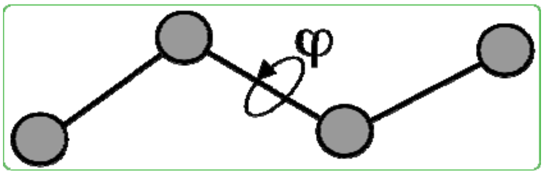

Fig S8: The dihedral angle φ is defined for four atoms bound by three bonds.

Flexibility of p51 residue 395

Minimal changes in the p51 residue 395 sidechain χ_1 dihedral angle are observed between the wild-type and A400T sequences in any of the four simulated states (see Fig S9). Fig S10 shows that the χ_2 dihedral angle shows greater variability in the wild-type sequence in all four systems.

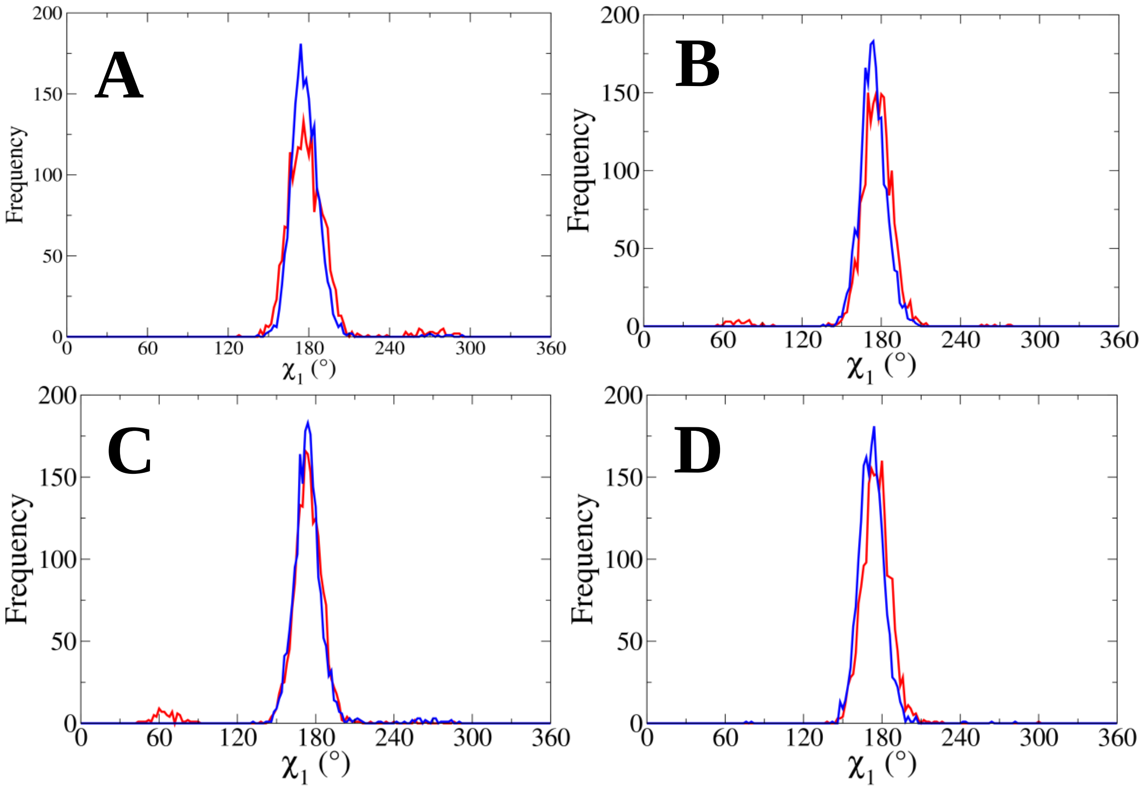

Fig S9: Distribution of the χ_1 sidechain dihedral angle of p51 residue 395 in simulations of HIV-1 RT (A) in the unbound state, (B) bound to NVP, (C) bound to RNA/DNA template/primer complex and (D) dsDNA template/primer. Results for the wild-type RT and for the A400T RT in blue.

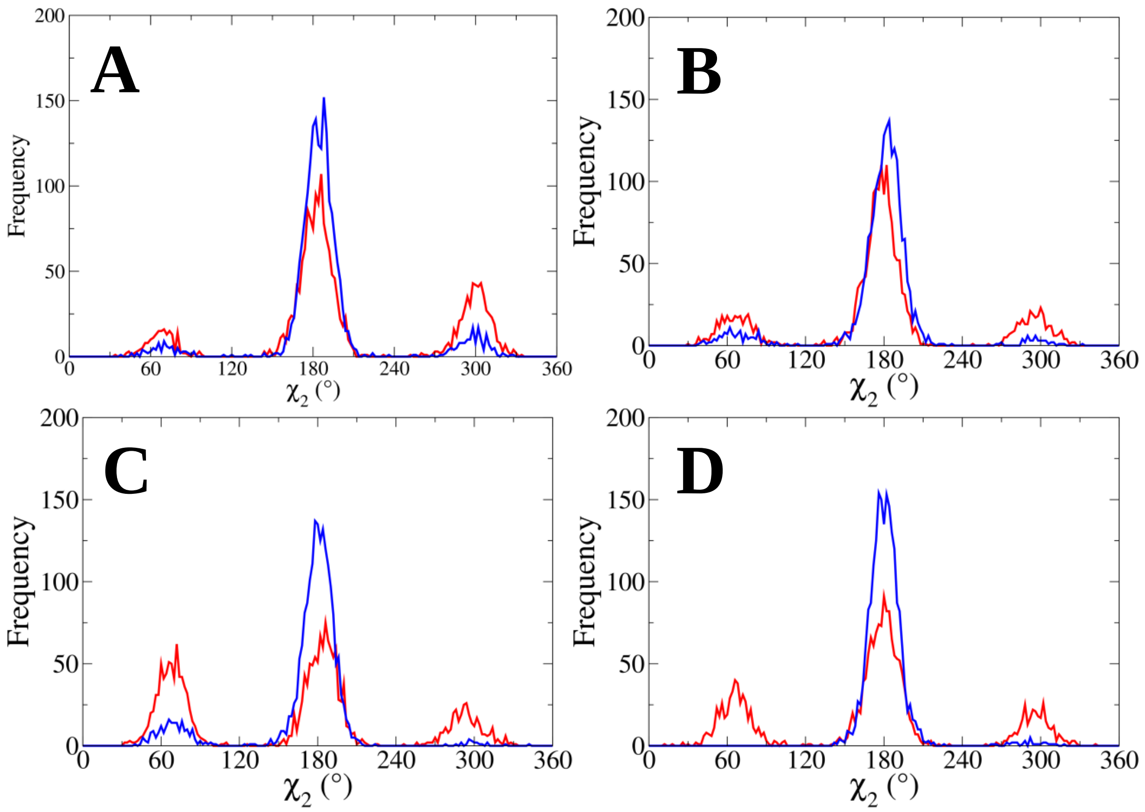

Fig S10: Distribution of the χ_2 sidechain dihedral angle of p51 residue 395 in simulations of HIV-1 RT (A) in the unbound state, (B) bound to NVP, (C) bound to RNA/DNA template/primer complex and (D) dsDNA template/primer. Results for the wild-type RT are shown in red, those for the A400T RT in blue.

Definition of the angle between p51 and p66 elements of the RNaseH primer grip

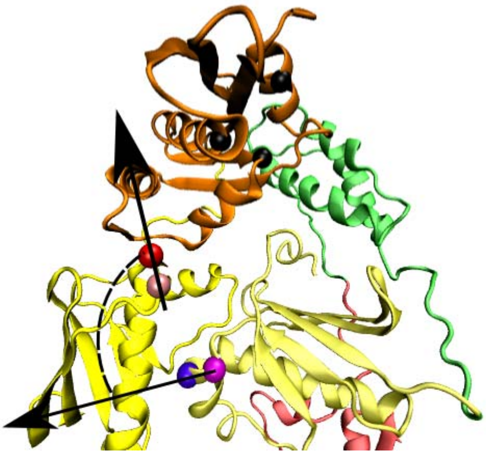

Fig S11: The angle between the RNaseH primer grip residues in p51 and p66. The location of the carbon alpha atoms of residues 395 (magenta) and 396 (purple) in p51and 359 (pink) and 360 (red) in p66. Vectors between these two pairs and the angle between them are shown in black.

Template flexibility

Fig S12 shows that, in line with experimental evidence [16], the RNA template in the wild-type HIV-RT is seen to show high flexibility around the p51 RNaseH primer grip residues, this flexibility is suppressed in the A400T RT. These results may be artificially exaggerated as the dsDNA template primer is comparatively short (18 nt compared to 29 nt in the hybrid). Fig S13 also shows that the dsDNA is held much more rigidly with low RMSF along both template and primer strands.

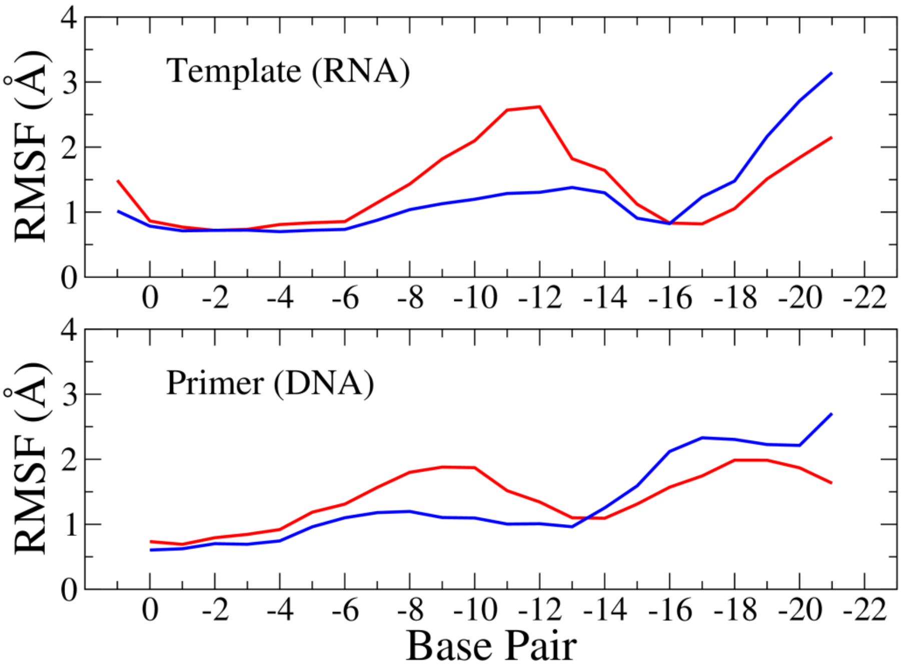

Fig S12: The RMSF along the RNA/DNA template/primer complex bound to the NL4.3 wild-type (red) and A400T (blue) HIV-1 RT. Base pair number is measured from the base interacting with the polymerase active site. The RNaseH primer grip residues p51 395 and 396 interact with bases -10 and -11.

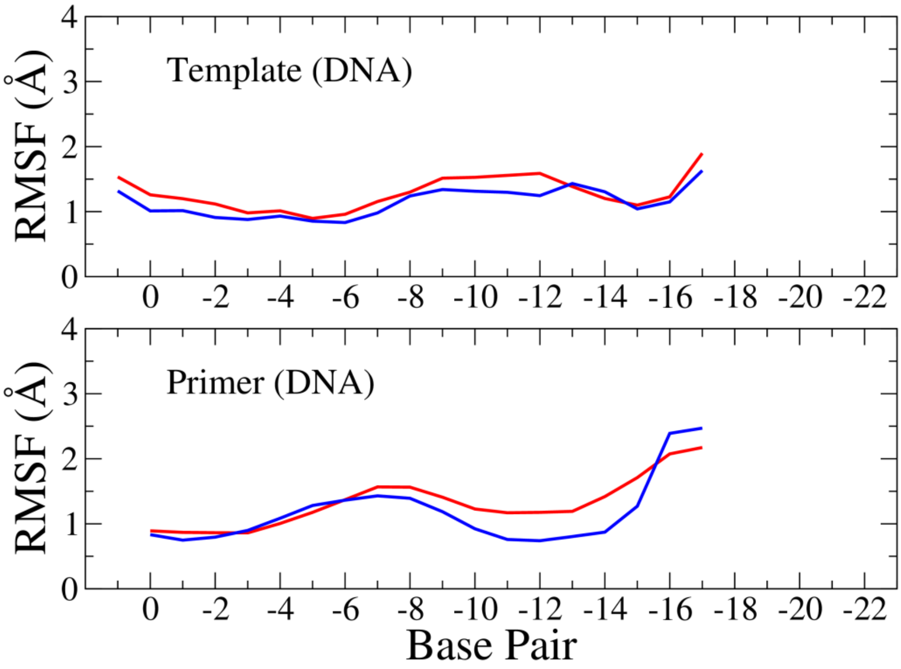

Fig S13: The RMSF along the dsDNA template/primer bound to the NL4.3 wild-type (red) and A400T (blue) HIV-1 RT. Base pair number is measured from the base interacting with the polymerase active site.

Structural changes in the template/primer

Differences in both the structure and flexibility of the RNA/DNA hybrid and dsDNA template/primer duplexes are seen between the wild-type and A400T RTs. It has been suggested that the recognition of template/primer duplexes is mediated by the minor grove width of the substrate [17, Biondi, #534]. Here we have calculated the minor groove width of the template/primer duplexes using the 3DNA package [18,19]. In the RNA/DNA template/primer complex differences in this property are seen as the duplex enters the RNaseH following interactions with the RNaseH primer grip residues p51 395 and 396 which interact with base pairs -10 and -11 (see Fig S14). Narrowing of the groove is thought to be necessary for RNaseH activity [17]. The minor groove is wider in the A400T simulation as it enters the RNaseH. The minor groove width is more dramatically altered in the dsDNA duplex (see Fig S15). However, none of the changes occur at the polymerase end of the

chain which is the only point which is involved in catalytic interactions as there is no RNA in this system which could be degraded.

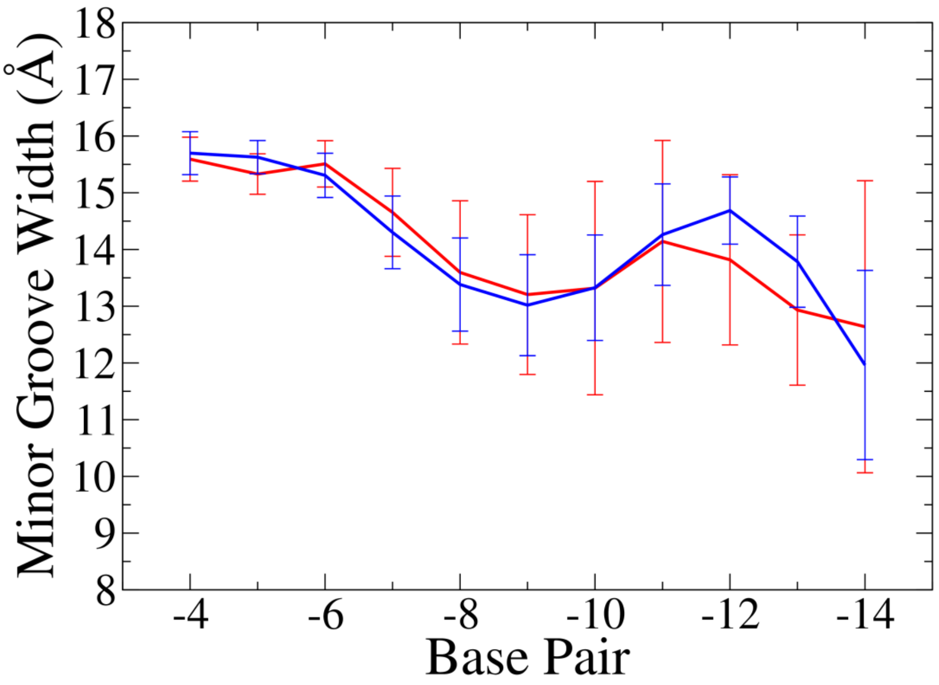

Fig S14: The minor groove width along the RNA/DNA template/primer complex bound to the N4.3 wild-type (red) and A400T (blue) HIV-1 RT. Base pair number is measured from the base interacting with the polymerase active site. The RNaseH primer grip residues p51 395 and 396 interact with bases -10 and -11.

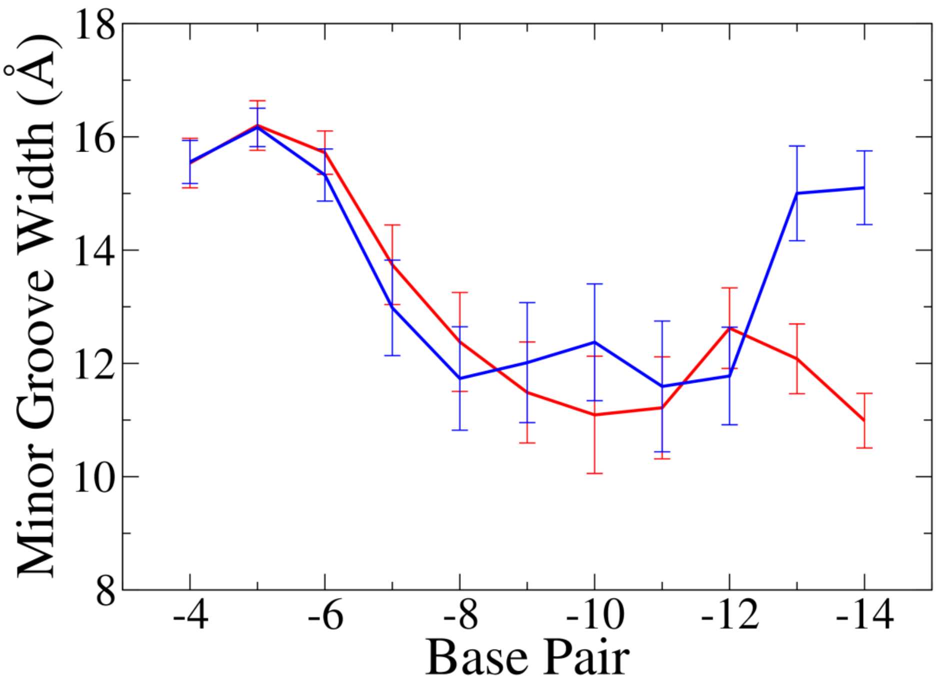

Fig S15: The minor groove width along the dsDNA template/primer bound to the wild-type (red) and A400T (blue) HIV-1 RT. Base pair number is measured from the base interacting with the polymerase active site.

Flexibility of p51 396 in the unbound enzyme

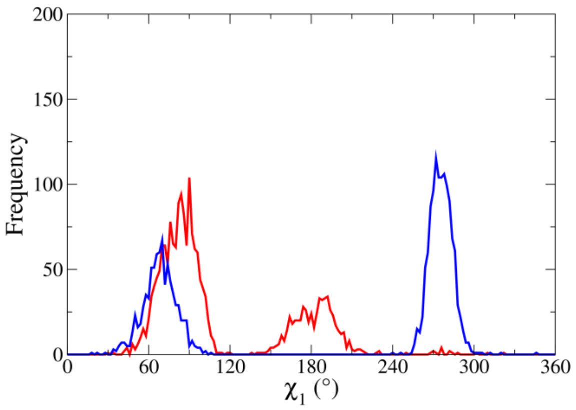

Fig S16: Distribution of the χ_1 sidechain dihedral angle of p51 residue 396 in simulations of unbound HIV-1 RT. Results for the wild-type RT sequence are shown in red, those for the A400T RT in blue.

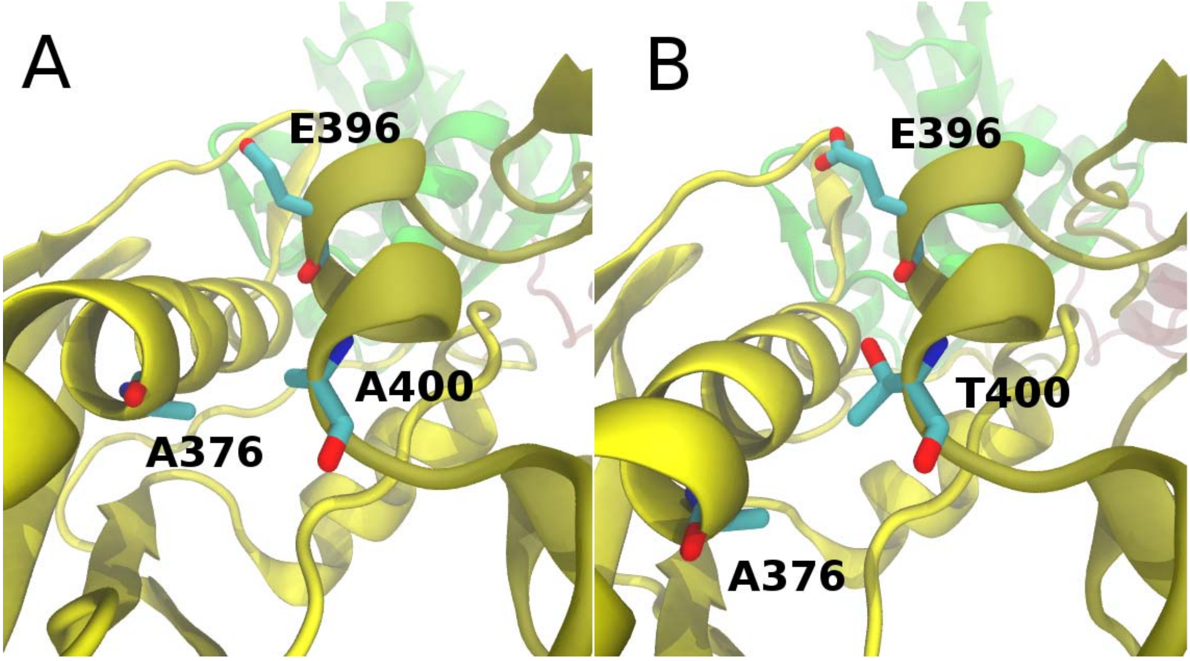

Fig S17: Average structures of the positions of the heavy atoms of residues 376 and 400 in the (A) wild-type and (B) A400T HIV-1 RT bound to DNA. The two residues pack more tightly in the wild-type RT that contains alanine residues at positions 400 and 376.

References

1. Humphrey W, Dalke A, Schulten K (1996) VMD: visual molecular dynamics. J Mol Graph 14: 33-38, 27-38.
2. Guex N, Peitsch MC (1997) SWISS-MODEL and the Swiss-PdbViewer: an environment for comparative protein modeling. Electrophoresis 18: 2714-2723.
3. Jorgensen WL, Chandrasekhar J, Madura JD, Impey RW, Klein ML (1983) Comparison of simple potential functions for simulating liquid water. The Journal of Chemical Physics 79: 926-935.
4. Schuttelkopf AW, van Aalten DM (2004) PRODRG: a tool for high-throughput crystallography of protein-ligand complexes. Acta Crystallogr D Biol Crystallogr 60: 1355-1363.
5. Frisch MJ, Trucks GW, Schlegel HB, Scuseria GE, Robb MA, et al. (2002) Gaussian 98.
6. Case DA, Cheatham TE, 3rd, Darden T, Gohlke H, Luo R, et al. (2005) The Amber biomolecular simulation programs. J Comput Chem 26: 1668-1688.
7. Wang J, Wolf RM, Caldwell JW, Kollman PA, Case DA (2004) Development and testing of a general amber force field. J Comput Chem 25: 1157-1174.
8. Duan Y, Wu C, Chowdhury S, Lee MC, Xiong G, et al. (2003) A point-charge force field for molecular mechanics simulations of proteins based on condensed-phase quantum mechanical calculations. J Comput Chem 24: 1999-2012.
9. Phillips JC, Braun R, Wang W, Gumbart J, Tajkhorshid E, et al. (2005) Scalable molecular dynamics with NAMD. J Comput Chem 26: 1781-1802.
10. Essmann U, Perera L, Berkowitz M, Darden T, Lee H, et al. (1995) A smooth particle mesh Ewald method. The Journal of Chemical Physics 103: 8577-8593.
11. Ryckaert JP, Ciccotti G, Berendsen HJC (1977) Numerical Integration of the Cartesian Equations of Motion of a System with Constraints: Molecular Dynamics of n-Alkanes. Journal of Computational Physics 23: 327.
12. Berendsen HJC, Postma JPM, van Gunsteren WF, Dinola A, Haak JR (1984) Molecular dynamics with coupling to an external bath. Journal of Chemical Physics 81: 3684-3690.
13. Sadiq SK, Wright D, Watson SJ, Zasada SJ, Stoica I, et al. (2008) Automated molecular simulation based binding affinity calculator for ligand-bound HIV-1 proteases. J Chem Inf Model 48: 1909-1919.
14. Zasada SJ, Coveney PV (2009) Virtualizing access to scientific applications with the Application Hosting Environment. Computer Physics Communications 180: 2513-2525.
15. Keller PA, Leach SP, Luu TT, Titmuss SJ, Griffith R (2000) Development of computational and graphical tools for analysis of movement and flexibility in large molecules. J Mol Graph Model 18: 235-241, 299.
16. Turner KB, Yi-Brunozzi HY, Brinson RG, Marino JP, Fabris D, et al. (2009) SHAMS: combining chemical modification of RNA with mass spectrometry to examine polypurine tract-containing RNA/DNA hybrids. RNA 15: 1605-1613.
17. Beilhartz GL, Gotte M HIV-1 Ribonuclease H: Structure, Catalytic Mechanism and Inhibitors. Viruses 2: 900-926.
18. Lu XJ, Olson WK (2008) 3DNA: a versatile, integrated software system for the analysis, rebuilding and visualization of three-dimensional nucleic-acid structures. Nat Protoc 3: 1213-1227.
19. Lu XJ, Olson WK (2003) 3DNA: a software package for the analysis, rebuilding and visualization of three-dimensional nucleic acid structures. Nucleic Acids Res 31: 5108-5121.
